# Reciprocal Inhibition of Immunogenic Performance in Mice of Two Potent DNA Immunogens Targeting HCV-Related Liver Cancer

**DOI:** 10.3390/microorganisms9051073

**Published:** 2021-05-17

**Authors:** Juris Jansons, Dace Skrastina, Alisa Kurlanda, Stefan Petkov, Darya Avdoshina, Yulia Kuzmenko, Olga Krotova, Olga Trofimova, Ilya Gordeychuk, Irina Sominskaya, Maria Isaguliants

**Affiliations:** 1 Institute of Microbiology and Virology, Riga Stradins University, LV-1007 Riga, Latvia; lisa9271@gmail.com (A.K.); olj.trofimova@gmail.com (O.T.); maria.issagouliantis@rsu.lv (M.I.); 2 Latvian Biomedical Research and Study Centre, LV-1067 Riga, Latvia; daceskr@biomed.lu.lv (D.S.); irina@biomed.lu.lv (I.S.); 3 Department of Microbiology, Tumor and Cell Biology, Karolinska Institutet, 17177 Stockholm, Sweden; stefan.petkov@ki.se (S.P.); olga.a.krotova@gmail.com (O.K.); 4 Chumakov Federal Scientific Center for Research and Development of Immune-and Biological Products of Russian Academy of Sciences, 108819 Moscow, Russia; avdoshina_dv@chumakovs.su (D.A.); gordeychuk_iv@chumakovs.su (I.G.); 5 Engelhardt Institute of Molecular Biology, Russian Academy of Sciences, 119991 Moscow, Russia; kuzmenko-yulia@mail.ru; 6 Institute of Translational Medicine and Biotechnology, Sechenov Moscow State Medical University, 119991 Moscow, Russia

**Keywords:** hepatitis C virus, hepatocellular carcinoma, immunotherapy, multi-component DNA vaccine, nucleocapsid (core) protein, telomerase reverse transcriptase, eukaryotic expression, CD4+ and CD8+ T cell response, immune suppression, assays of reporter expression, induction of type I interferons

## Abstract

Chronic HCV infection and associated liver cancer impose a heavy burden on the healthcare system. Direct acting antivirals eliminate HCV, unless it is drug resistant, and partially reverse liver disease, but they cannot cure HCV-related cancer. A possible remedy could be a multi-component immunotherapeutic vaccine targeting both HCV-infected and malignant cells, but also those not infected with HCV. To meet this need we developed a two-component DNA vaccine based on the highly conserved core protein of HCV to target HCV-infected cells, and a renowned tumor-associated antigen telomerase reverse transcriptase (TERT) based on the rat TERT, to target malignant cells. Their synthetic genes were expression-optimized, and HCV core was truncated after aa 152 (Core152opt) to delete the domain interfering with immunogenicity. Core152opt and TERT DNA were highly immunogenic in BALB/c mice, inducing IFN-γ/IL-2/TNF-α response of CD4+ and CD8+ T cells. Additionally, DNA-immunization with TERT enhanced cellular immune response against luciferase encoded by a co-delivered plasmid (Luc DNA). However, DNA-immunization with Core152opt and TERT mix resulted in abrogation of immune response against both components. A loss of bioluminescence signal after co-delivery of TERT and Luc DNA into mice indicated that TERT affects the in vivo expression of luciferase directed by the immediate early cytomegalovirus and interferon-β promoters. Panel of mutant TERT variants was created and tested for their expression effects. TERT with deleted N-terminal nucleoli localization signal and mutations abrogating telomerase activity still suppressed the IFN-β driven Luc expression, while the inactivated reverse transcriptase domain of TERT and its analogue, enzymatically active HIV-1 reverse transcriptase, exerted only weak suppressive effects, implying that suppression relied on the presence of the full-length/nearly full-length TERT, but not its enzymatic activity. The effect(s) could be due to interference of the ectopically expressed xenogeneic rat TERT with biogenesis of mRNA, ribosomes and protein translation in murine cells, affecting the expression of immunogens. HCV core can aggravate this effect, leading to early apoptosis of co-expressing cells, preventing the induction of immune response.

## 1. Introduction

Over 160 million individuals have been infected by HCV worldwide. Only one out of five patients spontaneously clear HCV infection, while in the rest, chronic infection is established. Chronic infection progresses to liver fibrosis, cirrhosis and development of liver cancer, majorly, the hepatocellular carcinomas (HCC). CD4+ T cells targeting a broad array of class II epitopes are detected during acute infection, but in chronic infection become undetectable or dysfunctional. CD8+ T cells detected in the liver in chronic HCV patients are exhausted, they lose their effector functions and fail to differentiate, explaining inability of immune system to combat HCV in chronic infection [1]. Nowadays, hepatitis C can be effectively treated by direct acting antivirals (DAA). DAA treatment inhibits viral replication, and partially restores the innate and adaptive immune response to HCV, but it is unable to treat T cell exhaustion [1]. HCV-induced immunological impairment continues after the successful DAA treatment [2]. Additionally, neither spontaneous, nor DAA-driven clearance of HCV infection preclude reinfection, although the latter occurs with milder clinical manifestations and results in a spontaneous clearance [3,4]. This speaks for the absence of sterilizing anti-HCV immune response providing a complete long-term protection. These findings brought understanding that a preventive sterilizing HCV vaccine might not be achievable. Today, the main efforts are directed towards HCV vaccines which would reduce the overall rate of HCV-associated morbidity and mortality, prevent the establishment of chronic infection, reduce the rate of reinfection, as well as correct anti-HCV immune response, aiding treatment of complications including liver cancer.

Current HCV vaccines in clinical trials or progressing towards the trials are mostly genetic [5,6]. New trials of therapeutic HCV vaccine based on naked DNA and on recombinant viruses are ongoing (https://clinicaltrials.gov/ct2/show/NCT04318379 (accessed on 12 February 2021); https://clinicaltrials.gov/ct2/show/NCT02772003 (accessed on 12 February 2021). Immense progress in vaccine development achieved under SARS-CoV-2 pandemics vividly demonstrated the huge potential of RNA and DNA vaccines [7]. DNA vaccines are specifically cost-efficient, they do not require a cold chain, which makes them useful in global applications, as could be the therapeutic vaccination of patients with chronic HCV infection.

Meta-analysis of chimpanzee vaccine trial data showed that suppression of acute-phase virus replication was associated with potent T cell response, and that vaccines based on the structural proteins ensured significantly higher clearance rates than those based on the nonstructural HCV proteins [8]. Indeed, several candidate vaccines successfully tested in chronic HCV patients included structural HCV proteins and/or their components. Examples are synthetic peptides derived from conserved regions of core in IC41 [9], DNA encoding HCV core and envelope E1 and E2 proteins in CIGB-230 [10], and recombinant HCV core produced in yeast cells in GI-5005 [11]. Interestingly, they included HCV core, a protein with plentitude of adverse properties including interference with several metabolic pathways and gene regulation cascades, modulation of apoptosis, with potential to promote cell growth and immortalization, and regulate the immune response [12,13,14]. Despite the adverse properties of HCV core, Drane et al. showed that a candidate HCV vaccine based on the recombinant HCV core protein with ISCOMATRIX™ adjuvant was safe and immunogenic, inducing T cell response with cytokine production and production of antibodies in preclinical and clinical trials [15]. HCV core is highly conserved [16]. Furthermore, it is expressed by all HCV infected liver cells, also malignant, and promotes transformation [17,18,19,20]. Taken together, this supports the use of HCV core in a therapeutic vaccine to prevent morbidity and mortality related to chronic HCV infection, including liver cancer.

In the era of DAA, HCV infection may be successfully eliminated in almost every patient. DAA treatment improves the conditions of patients with liver cirrhosis and even HCC, but even if successful, it does not eliminate the risk of developing liver cancer [21,22]. Additionally, DAAs are ineffective against liver cancer when it is already established, they cannot eliminate tumor cells and/or revert the process of tumorigenesis. Treatment of HCV-related liver disease would benefit from combination of DAA with immunotherapy, including therapeutic vaccination, aiming to eliminate tumor cells. Altogether, this formulates a need in a multi-component vaccine targeting both HCV infected and malignant cells.

Practical, as well as economic, factors shaping vaccine development promote the development of multi-component vaccines to be delivered in “one shot”, with the “one shot” option preferred to the individual delivery of the components [23,24,25]. In the vast majority of cases, the administration of polyvalent DNA vaccines does not result in an impairment, but rather in an enhancement of the overall immune response and vaccine efficacy [26,27,28], also for candidate multi-gene DNA vaccines against HCV in both preclinical and clinical trials [26,29]. However, interferences among components with respect to level of expression, or local, or systemic immune response have also been described, requiring changes in co-immunization regimens [30,31,32,33].

Here, we aimed to develop a two-component DNA vaccine with one component (based on HCV core) to target HCV infected cells, while the other was to target tumor cells, independently of their HCV status. For the latter, we chose a well-known tumor-associated antigen considered as a promising candidate for cancer vaccines, telomerase reverse transcriptase (TERT) [34]. We have previously described the high immunogenicity of TERT in mice, and the significantly restricted growth of tumors expressing reverse transcriptase domain of TERT in mice. We attributed the latter to the capacity of TERT as an enzyme to in vivo synthesize short RNA and DNA/RNA hybrids which can trigger innate immune response against tumor cells artificially made to overexpress TERT [35], and also act as molecular adjuvants enhancing the immunogenicity of TERT. Hence, we reasoned that the addition of TERT DNA immunogen would not only target the prototype vaccine to tumor cells, but would also further enhance the immune response against HCV core. With this in mind, we launched a trial of the immunogenicity of the expression-optimized DNA encoding HCV core and its combination with DNA vaccine encoding TERT. The trial revealed the incompatibility of these components, with reciprocal suppression of both anti-TERT and anti-HCV core immune response, warranting a study of the underlying mechanisms. Combination of in vitro and in vivo experiments indicated that (over)expression of TERT affected the process of transcription. It did not interfere with the immunogenicity of TERT if the latter was delivered alone, but completely suppressed it in the presence of HCV core, plausibly due to its pro-apoptotic activities. To circumvent this problem, one would need to deliver the components separately, or substitute HCV core with another HCV antigen expressed in chronically infected cancerous liver, or alternatively, design therapeutic vaccines against HCV-related HCC-based solely on TERT.

## 2. Materials and Methods

### 2.1. Plasmids

Plasmid directing expression of the full-length HCV core using the genomic sequence of HCV 1b isolate 274933RU (GeneBank accession #AF176573) based on eukaryotic expression vector pVax1 was described by us previously (pVaxCore191v; [36]). The sequence encoding HCV core aa 1-191 codon optimized for expression in mammalian cells, carrying flanking endonuclease restriction sites *Hind*III and *Xho*I, was synthesized by Epoch Life Science Inc. (Missouri City, TX, USA), cloned into SmaI-digested pBluescript II SK(-) and further recloned into pVax1 generating pVaxCore191opt. DNA encoding aa 1-152 of HCV core was amplified from pVaxCore191opt using oligonucleotides 5′-GCTTAAGCTTGCCGCCACCATGGACATGA-3′ as forward and 5′-CTAGACTCGAGCTATCAGGCCAGGGCTCT-3′ as reverse primers; the PCR product was digested with endonucleases *Hind*III and *Xho*I, and ligated into the *Hind*III/*Xho*I-cleaved pVax1 resulting in the plasmid pVaxCore152opt. The design of the pVax1-based vector for eukaryotic expression of rat telomerase reverse transcriptase (UniProtKB database accession number Q673L6) pVaxTERT (GenBank submission MK749423) was described previously [35]. The latter was subjected to site-directed mutagenesis to delete amino acid residues constituting the active center (aa 860-862, VDD) and the N-terminal 15 amino acid residues constituting the nucleolar localization signal NoLS [37] generating plasmid pVaxTERTin. The nucleotide sequence encoding the reverse transcriptase domain of rat TERT (rtTERT) was amplified from the prokaryotic expression vector pET15rtTERT [35] (Jansons J, 2020). Met-Gly dipeptide was added to the N-terminal residue of rtTERT. Together with the insertion of an ATT triplet upstream of the AUG codon, this introduced the consensus Kozak’s sequence ANNATGG required for the efficient initiation of rtTERT gene translation. Resulting DNA was digested by BamHI, and EcoRI and cloned into BamHI/EcoRI-cleaved pVax1, to generate plasmid for eukaryotic expression of rtTERT, which was further subjected to site-directed mutagenesis to delete amino acid residues constituting the active center, resulting in pVaxrtTERTin. Plasmid overexpressing enzymatically active RT (RThiv(a)) of HIV-1 clade B isolated from patient J14562 pVaxRT1.14opt(a) was described by us previously [38]. Enzymatic activity of RT was abrogated by point mutations D187N, D188N, and E480Q, introduced into the RT gene by site-directed mutagenesis [38], resulting in the plasmid pVaxRT1.14opt(in) encoding RT variant RThiv(in). The gene for RNA-dependent RNA-polymerase of HCV (NS5B) was amplified from the plasmid pI341/NS3-3′/LucUbiNeo-ET (HCV isolate Con1, GenBank: AJ238799); cleaved DNA fragment was re-cloned into pcDNA3.1(+) vector (Invitrogen) to generate pcDNA-NS5B (for details, see [39]). Gene expression after DNA immunization was assessed using a plasmid encoding firefly luciferase (Luc) pVaxLuc2 (kind gift of A.K. Roos, Karolinska Institutet, Stockholm, Sweden). In vitro and in vivo surveys of Luc expression under the control of human IFN-β promoter were performed using plasmid IFN-Beta_pGL3 (https://www.addgene.org/102597/) (accessed on 15 April 2020) [40]. Plasmids were produced in *E. coli* and purified using Plasmid EndoFree Kits (Qiagen, Hilden, Germany) as recommended by the manufacturer.

### 2.2. Synthetic Peptides

HCV core- and TERT-derived synthetic peptides, purified by HPLC to 70% with structure confirmed by mass spectrometry, were provided by SynPep Ltd. (Shanghai, China). The list of synthetic peptides used is given in Supplementary Table S1.

### 2.3. Analysis of Expression of HCV Core

Huh7 cells were seeded into 12-well plates in an amount of 2 × 10^5^ cells/well. The next day, cells were transfected with 0.5 ug of plasmid DNA using 1 µL Lipofectamine LTX and 0.5 µL Plus Reagent (both Invitrogen, Thermo Fisher Scientific, Waltham, Massachusetts, USA). Two days after transfection, cells were harvested, lysed in Laemmli buffer and boiled for 10 min. Lysates were loaded onto 16% SDS-PAAG and transferred onto nitrocellulose membranes (Thermo Fisher Scientific). Preblocked (1 h at room temperature with 5% nonfat milk in PBS) membranes were incubated with primary rabbit anti-core serum #93 diluted 1:5000 [41] at 4 °C overnight followed by a protein A horseradish peroxidase-conjugated antibody diluted 1:1000 (Thermo Fisher Scientific). In between incubations, membranes were washed twice for 10 min with PBS containing 0.5% Tween-20. Detection was performed with the DAB Substrate Kit (Thermo Fisher Scientific) according to the manufacturer’s protocol. After HCV core detection, blots were striped according to the detection system protocols and re-stained for signal normalization with mouse monoclonal anti-actin antibodies (AC-74, Sigma-Aldrich, St. Louis, MO, USA) diluted 1:3000 followed by a protein A horseradish peroxidase-conjugated antibody as above. Immunoblots were scanned, and signals of the individual bands were quantified using the ImageJ software (http://rsb.info.nih.gov/ij) (accessed on 12 February 2021).

### 2.4. In Vitro Luciferase Assay

HEK293 cells were seeded into 24-well plates in an amount of 1 × 10^5^ cells/well. The next day, cells were transfected with 0.5 µg of plasmid IFN-Beta_pGL3 (Addgene) encoding luciferase under the control of IFN-β promoter and 0.5 µg of either plasmids encoding HCV NS5B, or HIV-1 RT, or inactivated HIV-1 RT, or empty vector pVax1 using 1 µL Lipofectamine LTX and 0.5 µL Plus Reagent. Twenty hours post transfection, cells were harvested, lysed using RLB buffer (Promega, Madison, WI, USA), centrifuged, and supernatant was assessed for luciferase activity using the Luciferase Assay System (Promega) as recommended by the manufacturer. Luminescence was measured on a luminometer (Promega).

### 2.5. DNA Immunization

Two series of immunization experiments were performed. In the first, BALB/c mice were primed with two 20 µg doses of either pVaxCore191v (*n* = 5), or pVaxCore152opt (*n* = 5), or vector plasmid pVax1 (*n* = 5), and in the second, with pVaxTERT (*n* = 5); equimolar mixture of plasmids pVaxCore152opt and pVaxTERT (*n* = 5), or empty vector pVax1 (*n* = 5) (Table 1). In both, 3 weeks post prime mice were boosted with 15 µg of the same plasmid immunogens as used in prime, together with 5 µg of pVaxLuc2 (20 µg of DNA per site in total) (Table 1).

At each immunization, mice received two intradermal (id) injections of plasmid DNA solution in PBS delivered to the left and to the right from the back of the tail. Plasmids were administered with 29 G-needle insulin syringes. Injections were followed by electroporation using in vivo electroporator CUY21EditII (BEX Co., Tokyo, Japan) with fork-plate (CUY663-5 10) electrode (BEX Co., Tokyo, Japan) with a poration pulse of 400 V (0.1 ms with a 20 ms break) followed by eight altering polarity (+/−) driving pulses of 10 ms performed at 100 V with 20 ms intervals [42] (Latanova, Sci. Rep. 2018).

The experiment was terminated two weeks after the boost. The mice were humanely euthanized by cervical dislocation, spleens were excised and homogenized, and single cell cultures were prepared using nylon 70 µm cell strainers (Nunc, Roskilde, Denmark). ACK Lysing Buffer (Thermo Fisher) was used to remove the erythrocytes. Stocks of murine splenocytes were prepared in RPMI containing 50% fetal calf serum and 10% DMSO, frozen at −80 °C for 1 week, and then transferred to liquid nitrogen for later assessment.

### 2.6. Assessment of Cellular Immune Response

Cellular immune responses were assessed by multiparametric flow cytometry as described in [35] (Jansons, Bayurova Vaccines 2020). Splenocytes of mice immunized with HCV core variants, TERT and HCV core/TERT mixture and control vector-immunized mice were stimulated for 5 h in a CO^2^ incubator at 37 °C with solutions of TERT-derived peptides, pool of HCV core-derived peptides, luciferase-derived peptide (Table 1; 10 µg/mL) or with mitogens, phorbol 12-myristate 13-acetate (PMA) at 50 ng/mL in Series I, and a mix of PMA at 50 ng/mL and ionomycin at 1 µg/mL in Series II (both mitogens from Sigma-Aldrich, St. Louis, MO, USA) or medium alone in the presence of Golgi plug reagent (BD Pharmingen, Franklin Lakes, NJ, USA). After incubation, cells were stained for viability with the Fixable Viability Stain 660 (FVS660; BD Horizon #564405). Thereafter, cell surface staining was performed with a mixture of antibodies including FITC-conjugated anti-mouse CD8a (#553031) and APC-H7-conjugated anti-mouse CD4 (#560181). Cells were then washed, fixed, permeabilized using PerFix-nc Kit (Beckman Coulter, Brea, CA, USA), and stained with PE-conjugated anti-mouse IFN-γ antibodies (#557649), BV421-conjugated anti-mouse IL-2 antibodies (#562969), and BV510-conjugated anti-mouse TNF-α antibodies (#563386); all above antibodies were from BD Pharmingen. All stainings were performed in duplicates. In total, six staining runs were performed, with each run including one sample from each of three groups. Stained samples were analyzed on a FACSAria II cytometer (BD Biosciences, Franklin Lakes, NJ, USA).

Data were exported as FCS3.0 files using FACSuite software and analyzed using FlowJo X.07 program (FlowJo LLC, Ashland, DE, USA). First, the general lymphocyte population was defined, and viable cells were identified by the lack of FSV660 staining. From the viable population, cells of interest were defined by the expression of CD4 and CD8 surface markers and for production of cytokines IFN-γ, IL-2, and TNF-α. Data are presented as percent of CD4+ or CD8+ cells producing one, two, or three cytokines, from the total population of CD4+ or CD8+ cells. The percent of cells positive for IFN-γ, IL-2, and TNF-α after stimulation with growth medium (background) was subtracted from all values. Gating principles to generate inclusive subpopulations of lymphocytes expressing the IFN-γ, IL-2 and TNF-α cytokines are illustrated in Supplementary Figure S1. Specific populations of reactive cells were calculated by subtracting background response induced by incubation of cells in RPMI individually for each mouse. Boolean gating was used to generate exclusive subpopulations of lymphocytes expressing the IFN-γ, IL-2 and TNF-α cytokines in different combinations.

Quality of splenocytes obtained in two experiments was assessed by comparing response to mitogen, PMA. Stimulation with a mix of PMA and ionomycin tended to generate more responding CD4+ T cells than with PMA alone, but on the overall, no difference in the groups could be demonstrated indicating similar viability of cells after freezing-thawing (see example mitogen stimulated production of IL-2; Supplementary Figure S2A,B). For the assessment of immune response by flow cytometry at the experimental endpoint, we pooled splenocytes of two mice in Series II group II-2. Likewise, we pooled splenocytes of two mice in Series II group II-3. Flow cytometry data for these two groups is therefore represented by four instead of five entries.

### 2.7. In Vivo Imaging of Luciferase Gene Expression

Bioluminescence from the sites of injections of a mixture of DNA-immunogen or vector DNA and pVaxLuc2 was measured on days 1, 2, 5, 7, 9, and 12 after the boost by in vivo imaging (Spectrum; Perkin Elmer, Waltham, MA, USA), as described previously [35,42,43]. Prior to capturing of the luminescence signal, mice were injected intraperitoneally with a solution of XenoLight D-Luciferin potassium salt (Perkin Elmer) in PBS at a dose of 150 µg/g body weight. Ten minutes later, anesthesia was induced by 4% isoflurane and maintained by 2.5% isoflurane throughout the imaging procedure. Regions of interest (ROI) were localized around the injection sites, and the bioluminescence signal was quantified as the total photon flux (photons/s). Bioluminescence imaging data were processed using the Living Image^®^ software version 4.5 (Perkin Elmer).

### 2.8. In Vivo Promoter Activation Assay

Plasmids encoding TERT, inactivated TERT (TERTin), inactivated reverse transcriptase domain of TERT (rtTERTin), HIV-1 RT, HCV NS5B, or empty vector pVax1 were mixed 1:1 (molecular mass wise) with plasmid IFN-Beta_pGL3 and injected in a total amount of 20 µg into naïve BALB/c mice (*n* = 3 per DNA). Control animals were injected with 10 µg IFN-Beta_pGL3 (*n* = 4). DNA (DNA mixs) was administered as described for DNA immunization. Monitoring of bioluminescence from injection sites was performed directly after, and 24, 48, 72, 144 and 196 h post injection by in vivo BLI; data were acquired and processed as described above for bioluminescence imaging.

### 2.9. Ethical Statement

Experiments were carried in compliance with the bioethical principles adopted by the European Convention for the Protection of Vertebrate Animals Used for Experimental and Other Scientific Purposes (Strasbourg, 1986). Immunization experiments were approved by the Latvian Animal Protection Ethics Committee and the Latvian Food and Veterinary service, permit No 99 from 4 April 2018. In vivo reporter expression experiments were approved by the Northern Stockholm Ethical Committee for Animal Experiments (permit N66/13). Eight-week-old BALB/c mice purchased from Envigo (Venray, The Netherlands) or Charles River Laboratories (Sandhofer, Germany) were housed at a temperature of 22 °C under a 12-h light/dark cycle with ad libitum access to water and food. All animals were acclimatized for one week before starting the experiments. For the intradermal injections and electroporation, mice were anesthetized by a mixture of 4% isoflurane with oxygen and maintained in 2.5% isoflurane flow administered through facial masks or by ether inhaled from nose cones with soaked gauze.

### 2.10. Statistical Analysis

Continuous but not normally distributed variables, such as percent of cytokine producing CD4+ or CD8+ T cells, or photon flux, were compared using the nonparametric Kruskal-Wallis test and then pairwise using the Mann-Whitney *U* test with continuity correction. The Spearman rank-order correlation coefficient was calculated to characterize the linear correlations between variables. *p*-values < 0.05 were considered significant. Calculations were performed using Tibco Statistica 13.3 (Palo Alto, CA, USA).

## 3. Results

### 3.1. Design and Eukaryotic Expression of an Optimized HCV Core DNA Immunogen

**Figure 1:**
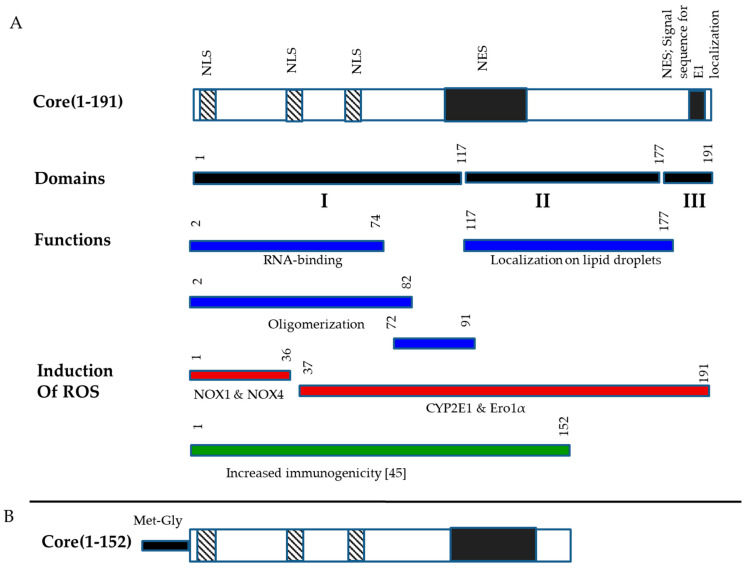
Structure of HCV core and its variant used for DNA immunization. Structural and structural domains of HCV core, domains responsible for RNA-binding, oligomerization, localization to lipid droplets, nuclear localization (NLS), nuclear export signals (NES), signal sequence for localization of E1 protein, induction of oxidative stress [13] and release of immunosuppression in in vivo tests [36] (**A**); Structure of HCV core aa 1-152 (Core152) encoded by expression optimized gene for the purpose of DNA immunization (**B**).

HCV core has several domains that interfere with viability of expressing cells, their metabolism, induction of innate immune response (Figure 1A). Specifically, we have earlier shown that C-terminal domain of HCV core interferes with HCV core immunogenicity [36]. We reasoned that the removal of C-terminus, the immunogenicity of the truncated variant can be further enhanced by increasing its expression level. Hence, we designed new optimized HCV core gene using codons frequently used in the mammalian cells (HCV Core191opt; GeneBank in deposition). Western blotting with polyclonal HCV core-specific antibodies showed expression in Huh7 cells of proteins with molecular mass of approximately 21 kDa corresponding to the full-length HCV core. The expression-optimized HCV Core191opt directed a five-fold higher level of protein expression compared to that of the viral gene (Supplementary Figure S3A–C). Furthermore, HCV Core191opt gene was modified by deletion of the fragment encoding the 39 C-terminal amino acids (Figure 1B). Plasmid pVaxCore152opt directed expression in Huh7 cells of the protein with the expected molecular mass of approximately 17 kDa corresponding to the truncated HCV core aa 1-152 (Core152opt with two aa residues on the N-terminus) (Supplementary Figure S3A,B).

### 3.2. Design of DNA Immunization Experiments

Immunization Series I aimed to assess immunogenicity of Core152opt DNA. Mice were DNA immunized with Core191v or Core152opt (Series I, Table 1). Series II assessed if anti-core immune response could be enhanced by delivering HCV core DNA together with DNA immunogen encoding telomerase reverse transcriptase (TERT DNA), so mice received plasmids encoding Core152opt + TERT; or TERT alone (Table 1). A group of mice receiving TERT DNA alone was included to assess possible effects of HCV core on anti-TERT immune response. Earlier studies indicated that introduction of the full-length HCV core gene can hamper immune response against co-delivered DNA immunogen [44,45]. Hence, we wanted to check if the C-terminal truncation prevents such suppression. In both series control animals received empty vector pVax1 (Table 1). DNA injections were followed by electroporation with strictly controlled electric current using an optimized prime/boost regiment described by us earlier [42].

In boosts, DNA-immunogens were supplemented with plasmid encoding firefly luciferase (pVaxLuc2; Table 1). We have earlier demonstrated that immune response against DNA immunogen induced in prime efficiently clears immunogen/reporter (luciferase) co-expressing cells after the boost, leading to a rapid loss of bioluminescence signal from the sites of immunization recorded by in vivo imaging [42]. We used this approach, dubbed “surrogate (antigen) challenge”, to assess the integral immune response induced by each of the immunogens and their combination as compared to the empty vector.

The endpoint immune response was assessed by flow cytometry assessing the percentage of CD4+ and CD8+ T cells responding to DNA immunogens by production of IFN-γ, IL-2 and TNF-α after stimulation with synthetic peptides derived from each of the immunogens. For this, we have selected a panel of peptides derived from HCV core and TERT shown to represent their immunodominant epitopes recognized in mice [35,36]. Peptides were used alone or in pools (Supplementary Table S1). We also included a peptide representing an immunodominant T cell epitope of luciferase [46] (LucP, Supplementary Table S1). Plasmid encoding Luc was injected once, in the boost (in “antigen challenge”). We have previously shown it to induce a weak cellular immune response already after one DNA immunization [47]. Hence, anti-LucP response could be used as a control of the quality of DNA immunization to ensure that it was performed similarly in all groups.

### 3.3. Cellular Immune Response against HCV Core

**Figure 2:**
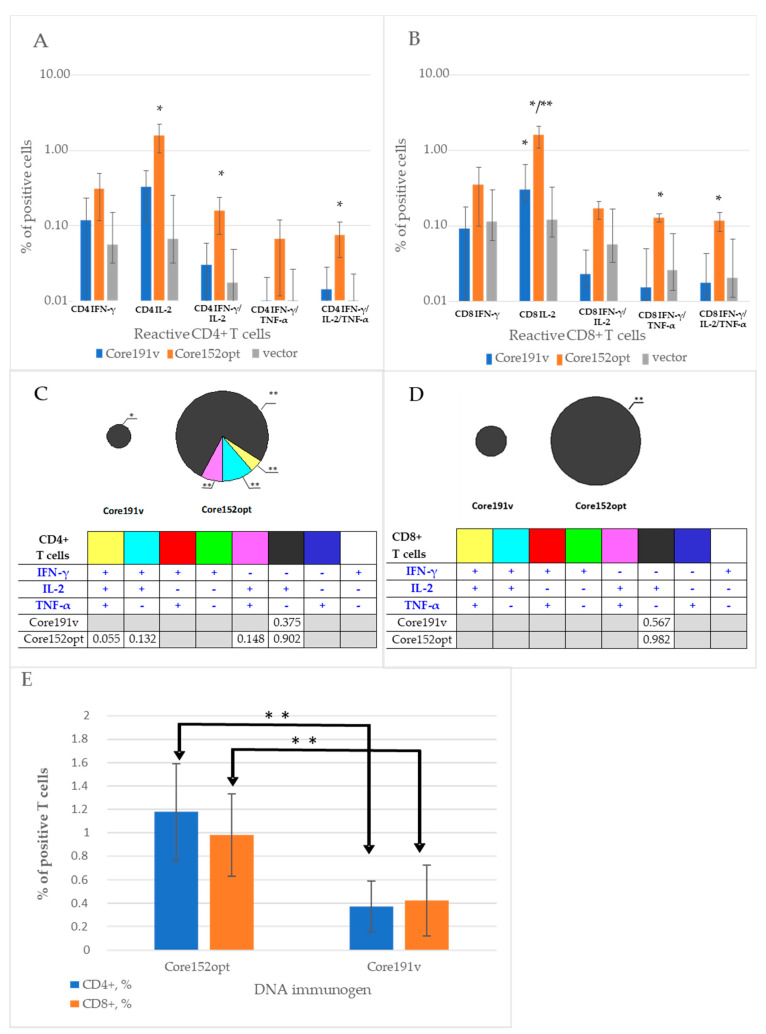
Specific T cell response in mice DNA immunized with Core191v or Core152opt compared to empty vector. CD4+ (**A**,**C**) and CD8+ (**B**,**D**) T cell response by inclusive (**A**,**C**) and exclusive (**B**,**D**) principles (exclusive visualize populations of cells secreting just the indicated cytokine), and total percent of reactive CD4+ and CD8+ T cells (**E**). Data from immunization series I; for vector mice, combined data from series I and II (Table 1). In panels (**A**,**B**,**E**), average per group ± STDV; in panels (**C**,**D**), average per group. Size of the pies is proportional to the total average % of T cells of a given type in the group. * *p* < 0.05; and (*) *p* < 0.1 in mice immunized with HCV core DNA compared to vector immunized animals; ** *p* < 0.05 in Core152opt versus Core191v immunized mice; pairwise comparisons by Mann-Whitney *U*-test.

In Series I, we assessed the immunogenicity of the expression optimized HCV Core152opt. Splenocytes collected by the experimental endpoint were assessed for the capacity to produce IFN-γ, IL-2 and TNF-α alone and in combination in response to stimulation with pool of peptides encompassing aa 61-175 of HCV core (Supplementary Table S1) recognized in mice [36,48]. In Core152opt DNA-immunized mice, we registered potent response characterized by secretion of IL-2, dual secretion of IFN-γ/TNF-α and triple secretion of IFN-γ/ IL-2/TNF-α by CD4+ T cells, while Core191v immunized mice responded by only by production of IFN-γ (Figure 2A,C). Pattern of cytokine production indicated lytic potential of the responding CD4+ cells indicated by their capacity to secrete IFN-γ, IL-2 and TNF-α (Figure 2). CD8+ T cell response in both groups was limited to mono production of IL-2, i.e., their profiles of CD8+ T cell response did not differ (Figure 2B,D). Magnitude of reactive CD4+ and CD8+ T cells in mice DNA immunized with Core152opt was two to three times higher than in mice receiving parental Core191v (Figure 2E).

### 3.4. Cellular Immune Response against TERT and TERT-HCV Core Combination

**Figure 3:**
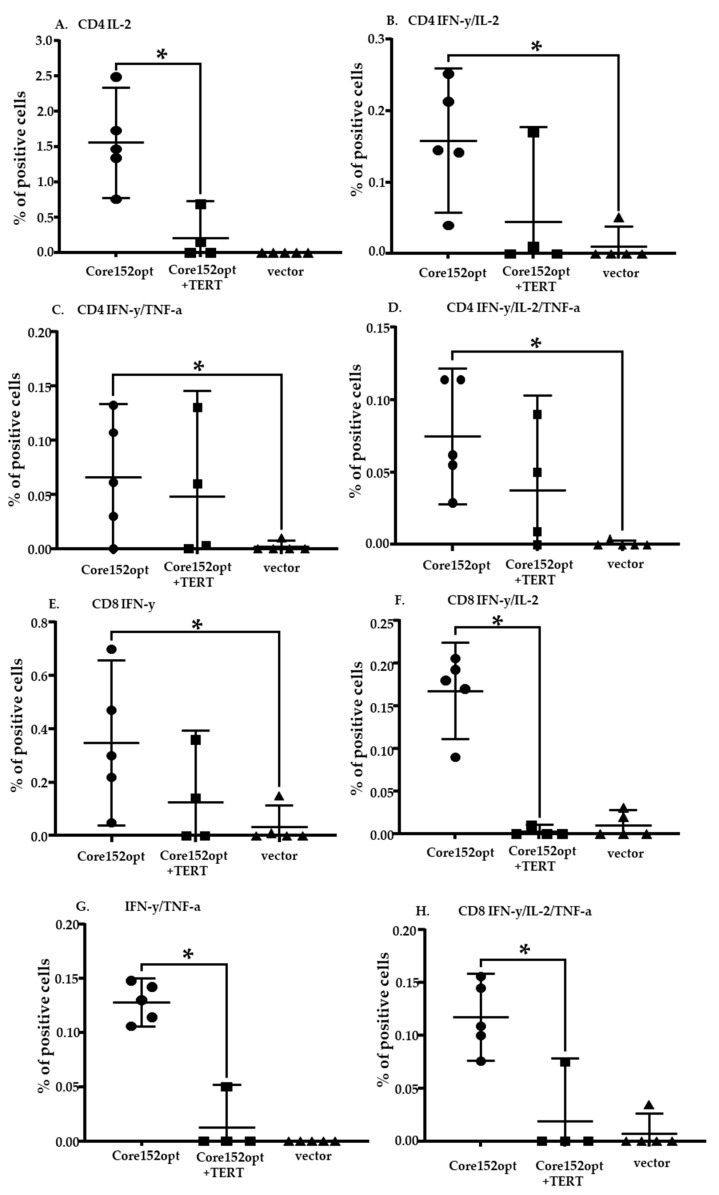
Cellular immune response against HCV core in mice DNA immunized with HCV Core152opt mixed with TERT compared to Core152opt alone (Series I and II, Table 1). Comparison of percent of CD4+ (**A**–**D**) and CD8+ (**E**–**H**) cells responding to stimulation with HCV core peptide pool (Table 1) by production of IL-2 (**A**,**E**), IFN-γ/IL-2 (**B**,**F**), IFN-γ/TNF-α (**C**,**G**), IFN-γ/IL-2/TNF-α (**D**,**H**). Data from is presented as mean ± 95% CI. Statistical analysis is made by Kruskal-Wallis and F-tests (Statistica Tibco, version 13.5): CD4+ T cells: IL-2: F(2,15) = 32.0616, *p* = 0.00000; KW-H(2,18) = 14.6055, *p* = 0.0007 (**A**); IFN-γ/IL-2: F(2,15) = 11.4882, *p* = 0.0009; KW-H(2,18) = 10.6863, *p* = 0.0048 (**B**); IFN-γ/TNF-α: F(2,15) = 4.9933, *p* = 0.0218; KW-H(2,18) = 8.4263, *p* = 0.0148 (**C**); IFN-γ/IL-2/TNF-α: F(2,15) = 12.4909, *p* = 0.0006; KW-H(2,18) = 12.6039, *p* = 0.0018 (**D**); CD8+ T cells: IL-2: F(2,15) = 30.9075, *p* = 0.00000; KW-H(2,18) = 12.6967, *p* = 0.0017 (**E**); IFN-γ/IL-2: F(2,15) = 22.7175, *p* = 0.00003; KW-H(2,18) = 11.1622, *p* = 0.0038 (**F**); IFN-γ/TNF-α: F(2,15) = 26.0782, *p* = 0.00001; KW-H(2,18) = 11.6205, *p* = 0.0030 (**G**); IFN-γ/IL-2/TNF-α: F(2,15) = 33.0762, *p* = 0.00000; KW-H(2,18) = 11.7138, *p* = 0.0029 (**H**). * *p* < 0.05., in pairwise comparison of mean values by Mann-Whitney test; *p* values are presented in Supplementary Tables S2 and S3.

Next, we proceeded to the assessment of T cell response induced by DNA immunization with a mixture of TERT and core encoding plasmids (dubbed MIX) (Table 1). Percent of CD4+ T cells secreting IFN-γ/ TNF-α and IFN-γ/IL-2/TNF-α in response to stimulation with core peptide pool in MIX-immunized mice tended to be higher than in mice receiving empty vector; however, the difference did not reach the level of significance (*p* = 0.07 and *p* = 0.05, respectively, Mann-Whitney *U* test; Supplementary Table S2). Other cell populations were indistinguishable from those in vector immunized mice (Supplementary Table S2). Further we compared MIX-immunized mice with mice DNA immunized with Core152opt (Supplementary Table S3; Mann-Whitney *U*-test, *p*-values <0.05 colored red, and *p* < 0.1, orange). Interestingly, the bulk of core-specific CD4+ T cells in MIX-immunized mice did not significantly differ from those in Core152opt-immunized mice (Figure 3B–D; Supplementary Table S3), confirming presence in these mice of core-reactive CD4+ T cells. The percentage of all core reactive CD8+ T cell populations in MIX-immunized was significantly lower than in mice DNA immunized with Core152opt except for mono IFN-γ producing cells (Figure 3E–H; Supplementary Table S3), demonstrating significant loss of HCV core-specific CD8+ T cell response. Thus, contrary to our expectations, DNA immunization with Core152opt/TERT mix did not boost the immune response against HCV core but resulted in its significant decline.

**Figure 4:**
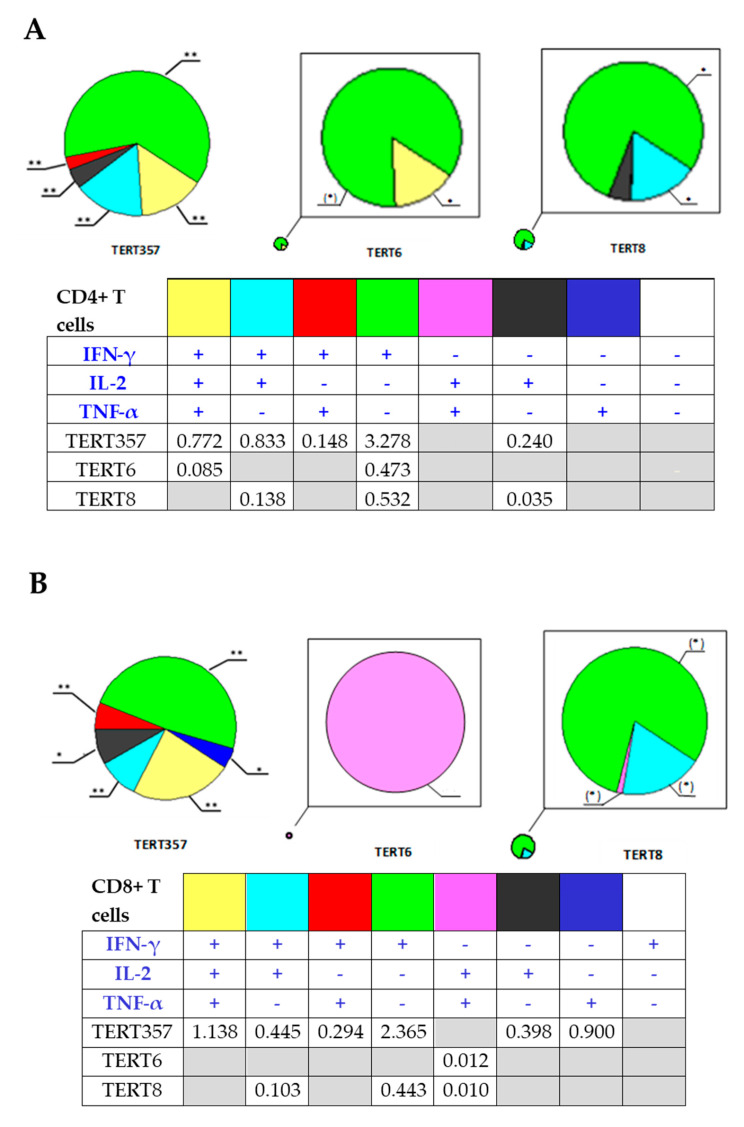
Structure of CD4+ and CD8+ T cell immune response against TERT induced by immunization with pVaxTERT plasmid. Average percent of CD4+ (**A**) and CD8+ (**B**) cells responding to stimulation with peptide pool TERT357, TERT6 and TERT8 (Supplementary Table S1) by production of one, two or three cytokines, visualizing exclusive populations. Tables beneath the graphs show actual percent of the respective populations, of total CD4+ (**A**) or CD8+ T cells (**B**). Populations in which TERT DNA immunized mice tend to differ from vector immunized mice are depicted in grey. Immunization details are described in Materials and Methods and groups, in Table 1. *—*p* < 0.05, **—*p* < 0.05 and (*)—*p* < 0.1 in comparison with vector immunized mice; grey filled empty boxes refer to absence of the respective T cell population (*p* > 0.1 compared to vector immunized mice). Statistical analysis in pairwise comparisons by Mann-Whitney *U* test (Statistica Tibco, version 13.5).

**Figure 5:**
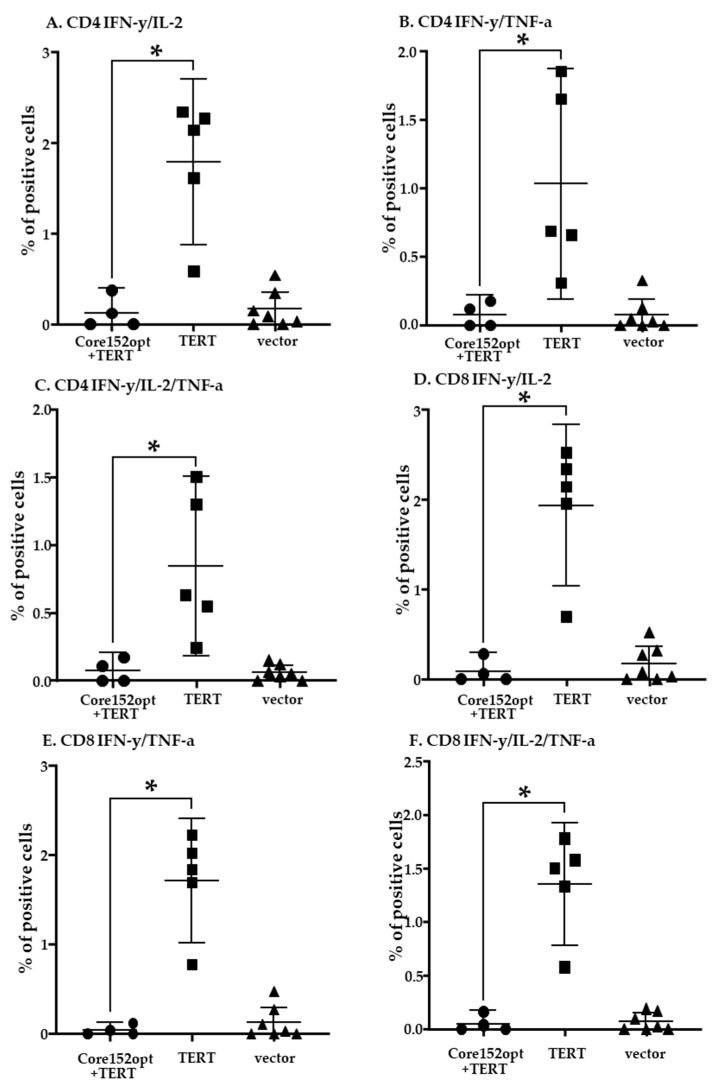
Cellular immune response against TERT in mice DNA immunized with TERT compared to TERT mixed with HCV Core152opt (Series II, Table 1). Comparison of percent of CD4+ (**A**–**C**) and CD8+ (**D**–**F**) cells responding to stimulation with peptide pool TERT357 (Supplementary Table S1) by production of IFN-γ/IL-2 (**A**,**D**), IFN-γ/TNF-α (**B**,**E**), IFN-γ/IL-2/TNF-α (**C**,**F**). Data from is presented as mean ± 95% CI. Statistical analysis is by Kruskal-Wallis and F-tests: CD4+ T cells: IFN-γ/IL-2: F(2,13) = 7.8551, *p* = 0.0058; KW-H(2,16) = 8.1131, *p* = 0.0173 (**A**); IFN-γ/TNF-α: F(2,13) = 3.8197, *p* = 0.0496; KW-H(2,16) = 7.4213, *p* = 0.0245 (**B**); IFN-γ/IL-2/TNF-α: F(2,13) = 4.1375, *p* = 0.0407; KW-H(2,16) = 6.734, *p* = 0.0345 (**C**); CD8+ T cells: IFN-γ/IL-2: F(2,13) = 7.0773, *p* = 0.0083; KW-H(2,16) = 7.7168, *p* = 0.0211 (**D**); IFN-γ/TNF-α: F(2,13) = 9.7326, *p* = 0.0026; KW-H(2,16) = 7.5156, *p* = 0.0233 (**E**); IFN-γ/IL-2/TNF-α: F(2,13) = 8.5704, *p* = 0.0042; KW-H(2,16) = 7.5156, *p* = 0.0233 (**F**). * *p* < 0,05 in pairwise comparison by Mann-Whitney test, for *p*-values see Supplemenatry Tables S4 and S5 (Statistica Tibco, version 13.5).

In view of the previously reported immunosuppressive properties of HCV core (demonstrated for the full-length protein [44,45]), we assessed TERT-specific immune response in mice receiving TERT alone and Core152opt/TERT mix. For this, we compared percent of CD4+ and CD8+ T cell populations recognizing individual and pooled TERT peptides harboring epitopes recognized in TERT-immunized mice [35]. In TERT DNA immunized mice, we detected a potent immune recognition of TERT357 pool by CD4+ and CD8+ T cells (Figure 4A,B; Supplementary Table S4). However, in MIX-immunized mice it was lost for both CD4+ and CD8+ T cells (Figure 5; Supplementary Table S5). Mice DNA immunized with TERT exhibited also weaker, but specific response of CD8+ T cells against TERT6 and CD4 + T cells against TERT8 (Supplementary Table S6). This response was also lost (Supplementary Table S7). Thus, combination of optimized HCV core and TERT DNA immunogens resulted in reciprocal inhibition of immune response against both components. the reason for the lack of adequate induction of immune response could be technical or biological, we proceeded to their analysis.

### 3.5. Assessment of the Quality of DNA Immunization and Immune Response in Mice Receiving HCV Core and TERT

**Figure 6:**
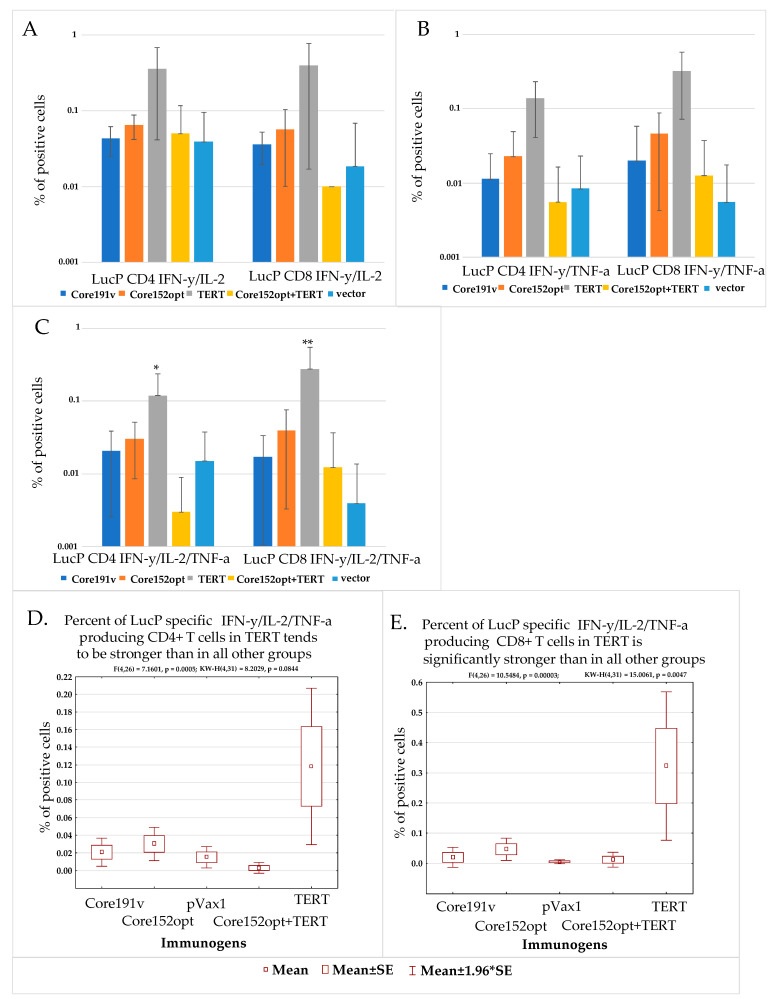
T cell response to immunodominant epitope at aa 166-168 of luciferase (LucP) in mice DNA immunized with Core191v, Core152opt, TERT, Core152opt/TERT mix or empty vector. Data from immunization series I and II; for vector mice, combined data from series I and II (Table 1). Percent CD4+ and CD8+ T cells secreting IFN-γ/IL-2 (**A**), IFN-γ/TNF-α (**B**), IFN-γ/IL-2/TNF-α (**C**); Statistical comparison of % IFN-γ/IL-2/TNF-α secreting CD4+ (**D**) and CD8+ T cells (**E**). Panels A-C, average ± STDV; D, E, mean ± standard error (ER). No difference between the groups in populations of IFN-γ/IL-2, and IFN-γ/TNF-α CD4+ and CD8+ in Kruskal Wallis test, and pairwise comparisons in Mann-Whitney *U*-test (**A**,**B**). ** *p* < 0.05, * *p* < 0.1, in pairwise comparison between mice DNA immunized with TERT versus other groups, Mann-Whitney *U* test (**C**) (Statistica Tibco, version 13.5).

Failure to induce the immune response can result from technical errors /inefficient immunization in this group. Quality of immunization in all experiments/groups could be assessed by monitoring percent of CD4+ and C8+ T cells responding to stimulation by peptide representing the immunodominant T-cell epitope of firefly luciferase (LucP, Supplementary Table S1). Luc induces weak CD4+ and CD8+ T cell response specific to LucP already after one DNA immunization [43,47]. With the exception of TERT DNA immunized mice, all groups, including mice co-immunized with Core152opt and TERT, demonstrated similar populations of dual (Figure 6A,B; Supplementary Figure S4) and triple cytokine responding CD4+ and CD8+ T cells specific to LucP (Figure 6C–E). This indicated that all immunizations (at least in boosts) were performed in a similar way with generation of comparable immune response. This spoke against technical faults as a reason for the low immune response against the main immunogens of the Core152opt/TERT plasmid mix. It also indicated that the suppression did not the response against Luc (weak as it was), affecting at the same time both anti-TERT and anti-core immune response (like a failure in both prime and boost in just this group).

Interestingly, we noted that percent of LucP responsive CD4+ and CD8+ T cells in TERT DNA-immunized mice was significantly higher than in all other groups (Supplementary Figure S4A,B,E,F; *p* < 0,05), specifically with respect to the population of IFN-γ/IL-2/TNF-α producing CD8+ T cells (Figure 6C). Populations of responsive T cells in other groups (excluding TERT immunized mice) did not differ (Supplementary Figure S4C,D,G,H; *p* > 0.05). This indicated that TERT alone did not preclude the induction of immune response against co-expressed protein(s) (here, luciferase encoded by the co-delivered reporter plasmid). On contrary, this response was enhanced, supporting our initial hypothesis of TERT “adjuvanticity” [35].

### 3.6. In Vivo Monitoring of Bioluminescence from the Sites of Injection of DNA Immunogens and Luciferase Reporter

**Figure 7:**
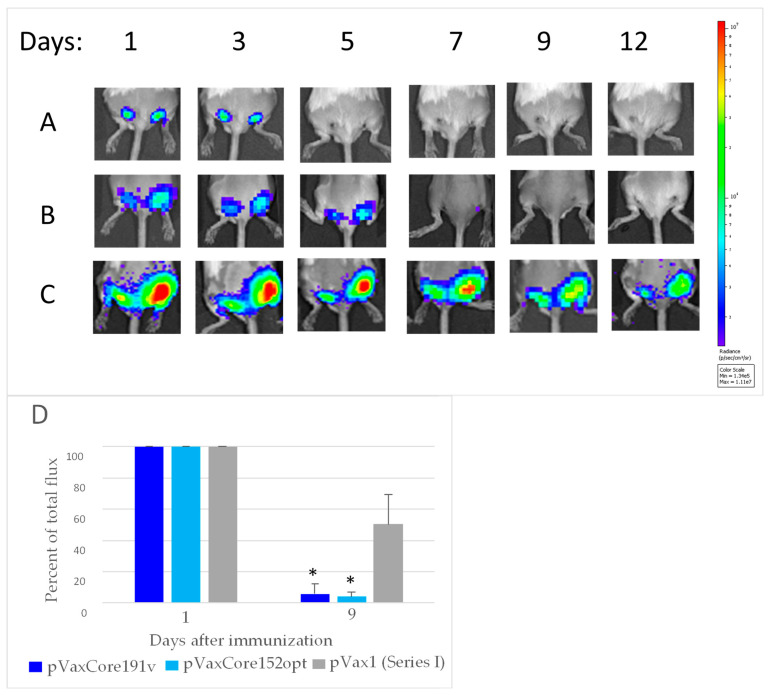
Loss of bioluminescence signal from the sites of boost with HCV core genes co-delivered with reporter plasmid encoding Luc. Mice were boosted with DNA encoding Core191v (**A**), Core152opt (**B**) or empty vector (**C**); average loss of BLI signal from day 1 to day 9, in % + STDV (**D**). Figures on the top show days after injections. Color scale to the right of the images in panels A-C represent signal intensity (photons/sec). * *p* < 0.05 between Core191v, Core152opt and vector immunized mice, F-test (Statistica Tibco 13.5).

One of the reasons of negative flow cytometry results for Core152opt/TERT DNA-immunized mice could be change of epitope dominance in mix immunization, requesting the choice of other peptides for screening. To check this option, we turned to our data from experiments on “antigen/surrogate” challenge testing the efficacy of immune response induced in prime. We boosted mice with the target DNA immunogens together with a plasmid encoding Luc reporter (Table 1), performing in vivo bioluminescence imaging (BLI) of the level of photon flux emitted from the injection sites from day 1 after the boost to the experimental endpoint. Data were expressed as percent of the maximal photon flux emitted from booster sites on days 1–2 post administration. Loss of BLI signal signified the immune clearance of cells co-expressing DNA-immunogen and reporter [42]. Indeed, by day 9 after the boost, bioluminescence signals from the injection sites of both core encoding plasmids were lost while most of the signal in the vector immunized mice was retained (Figure 7A–D). Loss of BLI signal due to immune clearance of immunogen/reporter co-expressing cells was indicated by tendency to inverse correlation of BLI signal with percent of IL-2 producing CD4+ and CD8+ T cells specific to HCV core peptides (Supplementary Table S1) (R = −0.4, *p* = 0.1, Spearman Rank Correlation test). Weakness of correlation could be attributed to the choice of peptides for screening. We have chosen peptides which we have shown to be well recognized in mice, but of different strain (C57Bl/6) [49]. Inclusion into the assessment of additional peptides (see, for example [50], IEDB Database) could have resulted in stronger correlations. Recombinant HCV core, which could have provided an opportunity to present all possible epitopes, was be used due to its immunosuppressive effect on the immune cells, also in in vitro tests [49].

**Figure 8:**
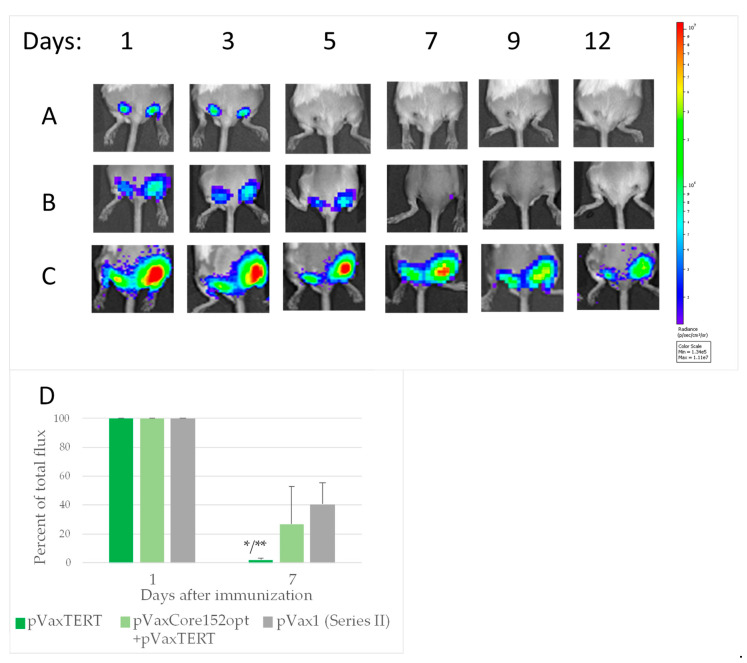
Loss of bioluminescence signal from the sites of booster DNA immunization with TERT, but not mixture TERT and core encoding plasmids. Mice immunized with plasmids encoding TERT (*n* = 5) (**A**), empty vector (*n* = 5) (**B**); mixture of plasmids encoding TERT and Core152opt (*n* = 5) (**C**), average percent loss from day 1 to day 7 (**D**). Images in panels A-C show individual mice followed in dynamics; figures on the top show days after DNA boost. Signal intensity in photons/sec is represented as a color scale to the right of the images. *—*p* < 0.05 as compared to vector immunized mice, ** *p* < 0.05, TERT versus Core152opt/TERT immunized mice, Mann-Whitney *U* test (Statistica Tibco, version 13.5).

In series II, similarly, a loss of BLI signal was observed after DNA immunization with TERT as compared to vector immunized mice, loss became highly significant by day 7 after the boost (Figure 8A,B; Supplementary Figure S5A–C). Much weaker loss was observed in mice co-immunized Core152opt/TERT plasmids (Figure 7C,D; Supplementary Figure S5A–C). BLI signal by day 7 inversely correlated with percent of CD4+ and CD8+ T cells specific to peptide pool TERT357 (Supplementary Table S1), specifically with the size of the population of IFN-γ/IL-2/TNF-α CD8+ T cells (IFN-γ/IL-2 CD4 + : r = −0.6152, *p* = 0.0333; IFN-γ/TNF-α CD4 + : r = −0.5908, *p* = 0.0431; IFN-γ/IL-2 CD8 + : r = −0.6674, *p* = 0.0177; IFN-γ/TNF-α CD8 + : r = −0.7198, *p* = 0.0083; IL-2/TNF-α CD8 + : r = −0.7123, *p* = 0.0093; IFN-γ/IL-2/TNF-α CD8 + : r = −0.7261, *p* = 0.0075; inclusive gating). These data signified immune clearance of expressing cells, serving as an integrate measure of specific immune response.

In summary, we detected a loss of BLI signal reflecting an “integrate” immune response against immunogen/reporter co-expressing cells in mice DNA immunized with Core191v, Core152opt and TERT, but not by Core152opt/TERT mix. Stable BLI signal backed up the flow cytometry data, and spoke of the reciprocal prohibition of immunogenic performance of HCV core and TERT, i.e., in favor of the third “expression conflict” scenario. Importantly, we also noted that mice DNA-immunized with TERT alone or mixed with Core152opt exhibited a decrease in BLI signal already on day 2 after the boost (Supplemantary Figure S5C,D).

### 3.7. In Vivo Assessment of the Effect of Co-Expression of Immunogens on the Initiation of Immune Response

Analysis of the expression of co-injected genes done on day 1 revealed that BLI signal in mice receiving TERT was significantly lower than in mice receiving HCV core variants or empty vector (Figure 8A; Supplementary Figure S5C,D; Supplementary Table S8). In Core152opt/TERT-immunized mice it tended to be even lower than in mice DNA immunized with TERT alone (Supplementary Table S8). This indicated a negative effect of TERT, specifically in the presence of HCV core, on the expression of Luc reporter. However, decrease in Luc activity/amount of protein did not affect anti-Luc response in the TERT group, on contrary, it was enhanced compared to all other groups (Figure 6C-E), indicating that this was the effect of TERT on reporter expression as such was not prohibitive for immune response against the reporter. Thus, the loss of immune response in Core152opt/TERT group could not be entirely attributed to the interference of TERT with the expression of (all) co-delivered genes.

We pursued this further and assessed if expression of TERT could modulate the following step, i.e., the induction of innate immune response. To this end, we used the bioluminescence reporter system in which Luc is placed under the control of human IFN-β promoter (IFN-Beta_pGL3 [40]), widely used in in vitro assays [51,52,53,54]. We reasoned that it could also function upon introduction in vivo, if the test and reporter plasmids enter one and the same cell, as was perfectly demonstrated in assessment of the in vivo transcriptional activity of NF-β [38,55]. In the pilot experiments, we tested the effect on the IFN-β promoter of the control proteins first in vitro, and then in vivo. As a positive control, we chose the RNA-dependent RNA-polymerase of HCV (NS5B), shown to induce expression of RIG-1, resulting in activation of expression from IFN-β promoter, and as controls related to TERT, enzymatically active and inactivated reverse transcriptases of HIV-1 RThiv(a) and RThiv(in), respectively. In vitro, plasmids were delivered by co-transfection, and in vivo, by intradermal injections followed by electroporation. In vivo pilot experiments demonstrated that introduction of an additional (even non-coding) DNA interferes with the early expression of the reporter (24 to 48 h post delivery; Supplementary Figure S6A–C). Hence, in further experiments, as a control we used the reporter plasmid mixed with the empty vector. Pilot experiments demonstrated that the positive control, HCV NS5B, effectively induced expression of Luc reporter both in vitro, and in vivo (Supplementary Figure S7A,B), demonstrating functionality of the assay.

**Figure 9:**
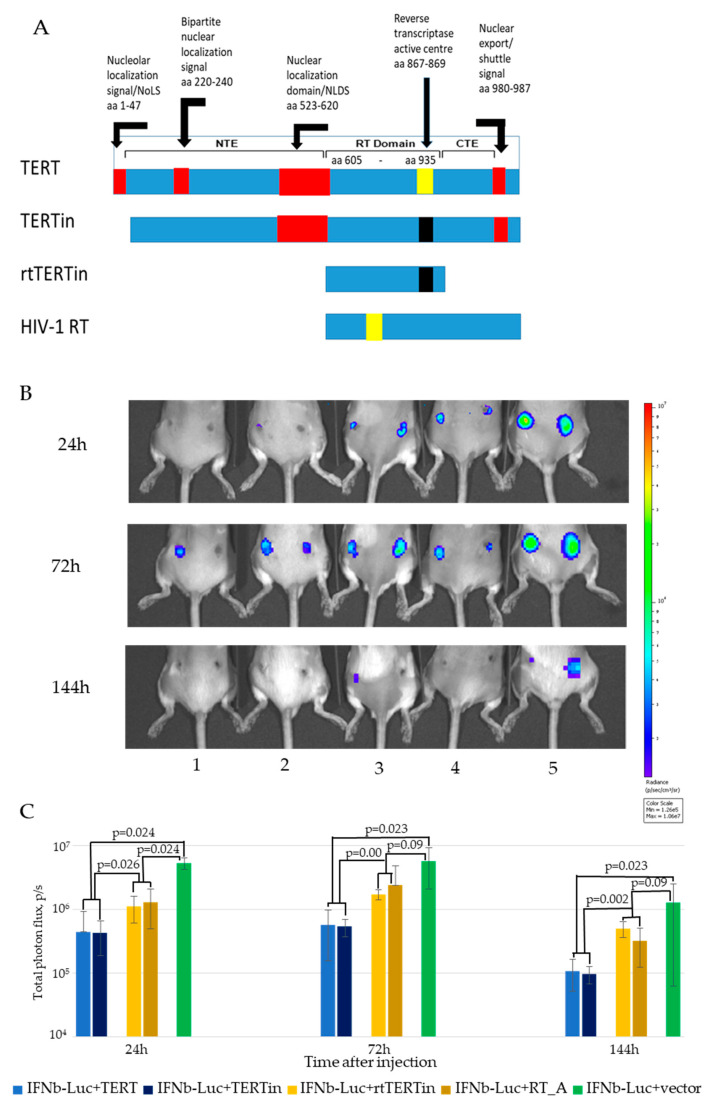
Enzymatically active TERT affects in vivo expression of luciferase reporter from a promoter of type I IFN gene. Reporter plasmid IFN-Beta_pGL3 directing synthesis of luciferase under the control of IFN-β promoter was co-injected into mice together with plasmids encoding Individual mice receiving injections of IFN-Beta_pGL3 (IFNbLuc) mixed with plasmids encoding active TERT (pVaxTERT), or inactivated TERT with deleted nucleolar localization signal and active center VDD (TERTin; pVaxTERTin), or its inactivated reverse transcriptase domain (rtTERTin; pVaxrtTERTin), or control protein, enzymatically active consensus reverse transcriptase of HIV-1 (RT_A; pVaxRThiv(a)) [38]. Schematic representation of the proteins, positions of signals and domains refers to human TERT (uniprot/O14746; positions of localization signals (red boxes), active center (yellow boxes) and mutated active center (black boxes) are indicated based on the publications [37,56,57,58,59] (**A**); sites of co-injections of IFN-Beta_pGL3 with pVaxTERT (1), or pVaxrtTERT (2), or pVaxTERTin (3), or pVaxRThiv(a) (4), or pVax1 (5) assessed 24, 72 and 144 h post injection (**B**); Level of photon flux from mice receiving the above injections (*n* = 3–4 per plasmid mix) (**C**). Color scale to the right from the images in panel B reflects signal intensity (photons/sec). Panel C, average level of photon flux ± STDV, pairwise comparison by Mann-Whitney *U* test.

In the main experiment, mice received IFN-Beta_pGL3 plasmid mixed with plasmids encoding enzymatically active TERT, or inactivated TERT with deleted nucleolar localization signal and mutated catalytic triad (TERTin), or the inactivated reverse transcriptase domain of TERT (rtTERTin), or enzymatically active HIV-1 reverse transcriptase RThiv(a). The best time points to assess modulation of expression were defined as days 1, 3 and 6 after the injection (Supplementary Figure S6C). In these settings we found that co-delivery of IFN-Beta_pGL3 with plasmids encoding both enzymatically active TERT and inactivated TERTin devoid of NoLS significantly inhibited the expression from IFN-β promoter from day 1 up-to the end of the follow up by day 6 (Figure 9C,D). The inhibitory effect of plasmids encoding rtTERTin and HIV-1 RT was significantly less pronounced, and could be reliably detected only during the first 24 h post injection (Figure 9C,D). With respect to HIV-1 RT, in vivo results reproduced those obtained in vitro in HEK293 cells showing weak inhibition of expression from IFN-β promoter independent of the enzymatic activity of HIV-1 RT (Supplementary Figure S7A). Thus, both active and inactivated HIV-1 RTs were found to cause mild suppression of IFN-β promoter activity in vitro tests.

Thus, TERT was found to affect the in vivo expression of co-delivered genes, including the expression driven by the IFN-β promoter in in vivo system modelling the induction of innate immune response. The findings indicated that TERT may shut off the expression of HCV core and, possibly, affect overall protein expression killing the expressing cells, and thus preclude the development of both anti-HCV core and anti-TERT immune response, as in the third scenario. This; however, contradicted our data on high immunogenicity of TERT (here and [35]) and its capacity to enhance the immune response against co-delivered reporter (Figure 6C–E), unless these processes occurred regardless of the negative effects of TERT. The latter implies a modified suppression scenario with HCV Core/TERT “conflict” in which TERT-driven immune response overcomes the negative effects of TERT, while HCV core abrogates this process resulting in a loss of immunogenicity of both components. Below we discuss these findings, possible mechanisms of the “conflict”, and its consequences for the development of multi-gene vaccine against HCV related HCC.

## 4. Discussion

To treat HCV-associated liver cancer, we proposed a bivalent DNA vaccine against HCV-associated liver cancer based on telomerase reverse transcriptase (TERT) and HCV core, and designed and optimized each of the components. HCV core is a highly conserved viral protein [16], expressed in all HCV infected cells, including those in the liver tumors [17,18,19]. We previously showed that plasmids inducing high level of expression of HCV core were less immunogenic than low-expressing vectors, even those missing the Kozak sequence [60]. In a later study, we attributed this to the capacity of the C-terminal domain of HCV core to induce production of ROS by activating cytochrome P450 2E1 (CYP2E1) [36]. HCV core DNA vaccine lacking the C-terminal CYP2E1 activating domain showed increased immunogenicity in mice [36]. We reasoned that truncation of the C-terminal domain would alleviate the immune suppression and set to enhance immunogenicity further by increasing the level of expression of truncated HCV core in mammalian cells [60]. An expression-optimized gene of HCV core aa 1-191 (Core191opt) was synthesized, which was expressed in mammalian cells at five times higher levels than the parental proteinCore191opt., and was truncated at the C-terminus generating HCV core aa 1-152 (Core152opt). DNA immunization with Core152opt induced potent cellular immune response, majorly of CD4+ T cells, significantly exceeding that against the parental gene. With this, we generated an optimized HCV-component of the bivalent vaccine.

TERT, a well-known tumor-associated antigen, is an enzyme responsible for the synthesis of telomeres, activated/overexpressed in many cancer cells. Enhanced telomerase activity allows cancer cells to replicate and proliferate in an uncontrolled manner, to infiltrate tissue, and to metastasize to distant organs. TERT is immunogenic, TERT-based immunogens easily overcome tolerance making TERT a perfect immunogen for cancer vaccines [34,61] making TERT an attractive target for cancer immunotherapy [34]. Continuous cell proliferation in the absence of sufficient telomerase activity causes extensive telomere shortening, leading to dysfunctional telomeres and genome instability by breakage–fusion–bridge cycles, which induce senescence or apoptosis as a tumor suppressor mechanism. Telomere shortening leads to reactivation of telomerase, promoting survival of tumor cells [62] and “TERT addiction” of liver tumors [63]. This makes TERT a specifically attractive target of therapeutic vaccines against liver cancer. We reasoned that a therapeutic vaccine against HCV-related HCC would benefit from the inclusion of the TERT component, making it effective against all malignant cells independently of their HCV infection status.

Different telomerase-targeting immunotherapies have been studied in preclinical and clinical settings [64]. DNA vaccines appear especially promising [65,66,67]. Recently, the TERT-based DNA vaccine INVAC-1 was evaluated in a phase I clinical trial (*n* = 26) in patients with advanced solid tumors. It induced CD8^+^ and CD4^+^ T-cell responses and, importantly, was able to reduce the numbers of circulating regulatory T cells at the same time increasing immune infiltrates into solid tumors and their metastases prompting further development of TERT-based cancer vaccines (https://clinicaltrials.gov/ct2/show/NCT02301754 accessed on 12 February 2021 [68]).

We based our TERT vaccine candidate on rat TERT, which we found to be highly immunogenic in mice [35]. Here, we confirmed that it induces potent CD4+ and CD8+ T cell response against multiple epitopes of TERT, which correlates with efficient clearance of TERT/reporter co-expressing cells from the site of immunization. Furthermore, we have shown that DNA immunization with TERT fully protects mice against challenge with TERT-expressing adenocarcinoma cells [69] (manuscript in preparation). We found TERT to be highly immunogenic, and, in the case of ectopic expression, capable of limiting the growth and metastatic activity of murine adenocarcinoma cells [35], and attributed these properties to the capacity of TERT to generate short RNAs and telomeric DNA/RNA hybrids [70,71,72] that mediate innate immune signaling [70,71,73], including the induction of type I IFNs. We hypothesized that co-delivery of TERT-based DNA immunogen would render additional stimuli for the development of immune response against HCV core. In support of this concept, in this study, we observed that co-administration of TERT DNA with Luc-encoding plasmid (as the reporter in “antigen/surrogate challenge” experiments) led to significant enhancement of CD4+ and CD8+ T cell response against luciferase, compared to the response in control animals receiving Luc DNA with empty vector. These experiments gave us the second component targeting tumor cells.

Having optimized HCV core and TERT DNA vaccine components, we launched the tests of their immunogenicity in a mixture. However, co-administration of DNA encoding Core152opt and TERT failed to enhance the immune response against HCV core, we registered barely detectable response by the specific CD4+ T cells. Furthermore, it led to complete loss of TERT specific immune response, i.e., we faced reciprocal prohibition of immune response against both components.

Plasmid interference was observed in multi-gene immunizations before [30,31,32]. Due to interference of the components, a multi-gene DNA vaccine against HIV-1 was administered as two plasmid cocktails delivered at spatially separate sites in preclinical, as well as clinical trials [33,74,75]. Mechanisms behind such interference are often not fully understood. Failure to induce immunity against multi-gene combination may result from one of the components being immunodominant, with consequent suppression of the immune response against other component(s) [76,77]. Components can also be directly immunosuppressive [31,78]. Neither of the reasons could explain our data, as both HCV core and TERT acted as strong T cell immunogens, and TERT also promoted an immune response against co-delivered luciferase, whereas HCV core/TERT combination resulted in a loss of the immune response against both components. Such suppression could be related to the competition on the level of expression [79]. Indeed, the presence of multiple copies of a functional CMV IE promoter in a noncoding (vector) plasmid can lead to a decrease in the expression of antigens encoded by a multivalent vaccine mixture [80]. Plasmid mixtures involving HCV core and reporter plasmids expressing IE CMV-controlled luciferase were used in all boosts, but did not reveal any differences in reporter expression directly after plasmid delivery. However, TERT was found to negatively impact in vivo expression of Luc from the IE CMV promoter, i.e., interference was not driven by the excess of DNA containing sequences of strong promoters, but was protein-specific.

We performed a series of experiments focusing on the effect of TERT on reporter expression (Figure 7 and Supplementary Figure S5C,D). The TERT gene used in the current study encodes native non-mutated rat telomerase reverse transcriptase to overcome tolerancein preclinical and eventual clinical trials. There are no safety requirements for TERT mutagenesis. Some of the TERT-based vaccines have no mutations except for the ones introduced to break tolerance [65]. Others are based on TERT with abrogated enzymatic activity alone or together with truncation of nuclear localization signal/NoLS [37]. Here, we applied the latter approach and designed full-length rat TERT lacking NoLS and catalytic center (TERTin). Besides, we have recently shown that the bulk of epitopes inducing lytic T cell response in TERT DNA immunized mice is localized in its reverse transcriptase domain, aa 605-935 in human, and 595-929 in rat TERT [35] (for the domain structure, see [81,82]). Expression of the rtTERT domain by murine adenocarcinoma cells drastically reduced their tumorigenic and metastatic activities [35]. The latter observation corroborated earlier findings in human cells: ectopic expression of the COOH-terminal fragment of the human TERT led to telomere dysfunction and reduction of growth and tumorigenicity of HeLa cells [83]. These findings motivated the use of rtTERT domain alone after its inactivation (rtTERTin).

The full-length TERT and two mutants TERTin devoid of the N-terminal NoLS and TERT active center, and rtTERTin containing only the reverse transcriptase domain with mutated active center, were assessed for the capacity to modulate the expression of type I IFNs, namely IFN-β, using the bioluminescence reporter system in which luciferase is placed under the control of human IFN-β promoter [40]. As control, we used enzymatically active or inactivated reverse transcriptase (RT) of HIV-1, viral protein related to rtTERT. Plasmid mixtures were delivered into mice, as was done during DNA immunization. Monitoring of reporter expression in this model allowed to assess possible modulatory effect on TERT on the expression as such as well as initiation of innate immune response. We found that co-delivery of both TERT and TERTin significantly inhibited reporter expression from the IFN-β promoter.The effect of rtTERTin did not differ from the effect of enzymatically active HIV-1 RT; both rendered significantly less effect on reporter expression than enzymatically active or inactivated TERT. These findings indicated that the effect of TERT on the presence of the full-length or nearly full-length protein was independent of its enzymatic activity, and supported the usefulness of TERT truncation up to the RT domain (while inactivation appeared to be optional).

We sought an explanation for the difference between the performance of TERT/TERTin and rtTERTin. Functions of TERT strongly rely on its localization; it is detected in the nucleolus—the site of ribosome biogenesis—in all phases of the cell cycle. In the nucleus, TERT has multiple partners. First in the relevance to the observed effects on transcription is nucleolin (NCL). NCL acts as a FACT-like protein (facilitates chromatin transcription), helping the passage of the RNA polymerase II through the nucleosomal particles. Global proteomics and interactomics approaches confirmed the prominent role of NCL in ribosome biogenesis and additionally revealed the possible involvement of nuclear NCL in several pre-mRNA processing pathways through its interaction with RNA helicases and proteins participating in pre-mRNA splicing, transport, or stability. NCL knockdown experiments revealed involvement of NCL in the control of mRNA stability [84]. Nucleolus controls the spatial dynamics and functions of NCL by affecting its subcellular localization. The absence of NCL from the fibrillar core of nucleolus, where it facilitates transcription, replication and recombination of rDNA, halts these early stages of ribosome biogenesis [85]. Interactions of xenogeneic (rat in relation to mouse host) ectopically overexpressed TERT with NCL can affect subcellular localization of NCL and interfere with its functions in the biogenesis of ribosomes.

Another set of TERT partners in the nucleus are AAA-ATPases, molecular engines driving the remodeling of proteins and macromolecular assemblies [82]. Their dysfunction disturbs the formation of functional ribosomes and lead to the defects in cell proliferation and growth [86]. Specific AAA-ATPase, valosin-containing protein-like 2 (NVL2) serves as an unfoldase for the nucleolin-RNA complex. As inferred from its RNA dependence and its ATPase activity, NVL2 might facilitate the dissociation and recycling of nucleolin, thereby promoting efficient ribosome biogenesis [87]. Disruption of NVL2 functions inhibits ribosome biosynthesis (dominant negative NVL2 mutants) [88]. Overexpression of TERT with relocation to the nucleoli and binding to AAA-ATPases, specifically to NVL2, may interfere with ribosome biogenesis, with adverse overall effects on the protein synthesis.

Among TERT partners in the nucleoli are the heterogeneous nuclear ribonucleoproteins (hnRNPs), RNA-binding proteins that participate in mRNA biogenesis in the nucleus and its subsequent translation in the cytoplasm [89]. HnRNPs assist in controlling the maturation of newly formed heterogeneous nuclear RNAs (hnRNAs/pre-mRNAs) into messenger RNAs (mRNAs), stabilize mRNA during their cellular transport and control their translation [90]. Of the several hnRNPs that are involved in telomere and telomerase, two—A2/B1 and A18—co-localize with TERT in the nucleolus [82]. Binding to TERT may critically reduce the number of available/functional of these hnRNP molecules, interfere with their capacity to regulate transcription, perform mRNA polyadenylation, and affect trafficking and/or stability of mRNA, i.e., drastically affect both transcription and translation in cells (over)expressing ectopically delivered TERT.

Last but not least, endogenous TERT, driven by mutant promoters or oncogenes, directly associates with the RNA polymerase III (pol III) subunit RPC32 and enhances its recruitment to chromatin, resulting in increased RNA pol III occupancy and expression of tRNA [91]. Xenogeneic ectopically overexpressed TERT may bind RPC32, but fail to enhance its recruitment to chromatin, interfering with translation. In summary, we hypothesize that ectopically expressed TERT may interfere with the functions of nuclear/nucleolar proteins and RNP critical for biogenesis of mRNA, ribosomes and/or translation, rendering systemic negative effects on transcription and translation The effects of xenogeneic TERT on protein biosynthesis and viability of (over)expressing cells requests a separate study.

TERT truncated up to the RT domain was devoid of the domains and signals of nuclear/nucleolar localization and shuttling except for the one preceding the reverse transcriptase domain remained intact [57] (see Figure 9A and references therein). This could have alleviated the above effects. However, negative effects of TERT on the reporter expression (and, possibly, cell viability) did not prevent TERT from being immunogenic, and did not preclude the immune response against co-delivered reporter. In this context, one can recall that surgical removal of the injected muscle within 10 min of injection of plasmid DNA did not affect the magnitude or longevity of the antibody response against the encoded immunogen; a short-term expression was sufficient to initiate an immune response [92]. Even if all TERT/Luc-co-expressing cells died within 24 h from DNA delivery due to ectopic overexpression of xenogeneic TERT, this did not preclude the development of anti-TERT or anti-Luc immune response, and was not expected to interfere with the immune response against HCV core. However, we registered a prohibition of the immune response against TERT. This implied a direct involvement of HCV core in the events occurring at the injection/expression site directly after DNA inoculation. Indeed, HCV core modulates apoptosis via diverse pathways relying on the multiple domains/motives within the protein. Some, such as that in the C-terminal region (residues 153 to 192) required for Fas ligand-independent apoptosis [93], are gone in the truncated HCV core aa 1-152, but others remain. HCV genotype 1b core aa 1 to 153 binds to the death domain of FADD, resulting in enhanced apoptosis [94]. Mohd-Ismail NK et al. identified a Bcl-2 homology 3 (BH3) domain at aa 115-129 of HCV 1b core, truncation of which abolished the induction of apoptosis [95]. Nuclear forms of HCV core are generated in vivo in primary hepatocytes and induce PKR-dependent apoptosis [96].

The net effect of HCV core would rely on the strength of these proapoptotic signals and other signals it communicates to the expressing cell. Ectopic overexpression of TERT could tap the balance, leading to the massive death of HCV core/TERT co-expressing cells. This would affect cells co-transfected with TERT and HCV core, and triple-transfected with TERT, HCV core and Luc encoding plasmids. The latter scenario is in line with the tendency towards higher loss of BLI signal from the sites of DNA boost with TERT and HCV Core152opt compared to TERT alone. As was shown in the oocytes of *Xenopus laevis*, apoptosis affects the cell in waves travelling at a speed of approximately 30 microns per minute [97]. For an ordinary eukaryotic cell with the size of 10 to 20 microns, this implies an immediate death, not leaving time for expression/accumulation of encoded proteins needed to trigger the immune response.

Both the effects of ectopic (over) expression of xenogeneic TERT on mRNA biogenesis, biosynthesis of ribosomes and protein translation, and of HCV core on cell viability/apoptosis, which we link to the reciprocal prohibition of HCV core and TERT in multi-gene DNA immunization, deserve a detailed mechanistic study.

## 5. Conclusions

Treatment of HCV-related liver cancer, mainly represented by hepatocellular carcinomas, would benefit from immunotherapy targeting malignant HCV-infected and non-infected, as well as non-malignant HCV infected, cells, especially in cases of HCV resistant to DAA. The SARS-CoV-2 epidemic has strongly promoted use of genetic vaccines, including ones using plasmid DNA. In the context of HCV-related liver cancer, this implies multi-gene DNA vaccines with components targeting HCV and HCC. Such multi-gene DNA vaccines against various pathogens have been developed, with many success stories, but there are also examples of negative interference. We developed two prototype DNA vaccines based on expression optimized synthetic genes, one for HCV, based on its nucleocapsid (core) protein, the other for HCC, based on telomerase reverse transcriptase of rat. Each performed as a strong immunogen in mice, inducing potent multi-cytokine response of CD4+ and CD8+ T cells against multiple epitopes in both proteins. The immune response could clear cells co-expressing HCV core or TERT and bioluminescence reporter protein from the sites of plasmid injections. However, delivery of HCV core and TERT encoding plasmids in a mixture abrogated the immune response against both proteins. Possible mechanism of interferences implies negative effects of ectopic overexpression of xenogeneic TERT on mRNA biogenesis/biosynthesis of ribosomes, interfering with expression of immunogens and induction of immune response. Co-expression of HCV core can aggravate this effect, inducing apoptosis of co-transfected cells. The interference of HCV core and TERT as DNA immunogens warrants further study, and calls for an alternative approach of sequential treatment of HCV by therapeutic immunization in combination with DAA, followed by therapeutic vaccination against tumor associated antigens, such as TERT.

## Figures and Tables

**Table 1:** DNA immunization experiments.

Group	Nn Mice	Prime		Boost	
		Plasmids	Dose per Injection/ Number of Injections	Plasmids	Dose per Injection/ Number of Injections
**Series I** I-1	5	pVaxCore191v	20 µg × 2	pVaxCore191v + pVaxLuc2 *	15 µg × 2
I-2	5	pVaxCore152opt	20 µg × 2	pVaxCore152opt + pVaxLuc2 *	15 µg × 2
I-3	5	pVax1	20 µg × 2	pVaxTERT + pVaxLuc2 *	15 µg × 2
**Series II** II-1	5	pVaxTERT	20 µg × 2	pVaxTERT + pVaxLuc2 *	15 µg × 2
II-2	5	pVaxTERT + I have pVaxCore152opt	10 µg each × 2	pVaxTERT + pVaxCore152opt + pVaxLuc2 *	7.5 µg each × 2
II-3	5	pVax1	20 µg × 2	pVax1 + pVaxLuc2 *	15 µg × 2

* pVaxLuc2, always 5 µg per injection, with total plasmid dose in a boost of 10 µg per animal.

## Data Availability

No applicable.

## Supplementary Materials

The following are available online at https://www.mdpi.com/article/10.3390/microorganisms9051073/s1, **Figure S1**. Principles of inclusive gating applied in the analysis of specific T-cell response in DNA immunized mice using multiparametric flow cytometry. Screenshots with diagrams from FlowJo program. **Figure S2**. Splenocytes of mice in experimental series I and II (Table 1) demonstrate efficient response to stimulation with mitogen(s). Screenshots from Tibco Statistics program. **Figure S3**. Expression of HCV core variants in transiently transfected Huh-7 cells. Scanned images of Western blots and diagram with results of quantification analysis. **Figure S4**. Statistical comparison of T cell response to immunodominant epitope of luciferase LucP in mice DNA immunized with Core191v, Core152opt, TERT, mixture of Core152opt and TERT and empty vector. **Figure S5**. Bioluminescence from DNA booster sites in mice receiving HCV core and TERT alone or in a mix. Diagram with quantification data, screenshots from BLI registration program, diagrams with statistical analysis. **Figure S6**. Expression in mice of luciferase reporter from the IFN-β promoter. Screenshots from BLI registration program, diagram with quantification data. **Figure S7**. Expression of luciferase reporter from the IFN-β promoter in vitro (**A**) and in vivo (**B**). Diagrams with quantification data. **Table S1**. Synthetic peptides used in the study. Abbreviations of peptides, aa sequences and positions according to TERT or HCV core proteins respectively. **Table S2**. Statistical comparison of populations CD4+ and CD8+ cells responding to stimulation with HCV core peptide pool (Supplementary Table S1) in mice DNA immunized with mixture of Core152opt and TERT (MIX) compared to vector pVax1 (Mann-Whitney *U* test w/ continuity correction). **Table S3**. Statistical comparison of populations CD4+ and CD8+ cells responding to stimulation with HCV core peptide pool (Supplementary Table S1) in mice DNA immunized with mixture of Core152opt and TERT (MIX) compared to Core152opt (Mann-Whitney *U* test w/ continuity correction). **Table S4**. Statistical comparison of populations CD4+ and CD8+ cells responding to stimulation with TERT357 peptide pool (Supplementary Table S1) in mice DNA immunized with TERT compared to vector pVax1 (Mann-Whitney *U* test w/ continuity correction). **Table S5**. Statistical comparison of populations CD4+ and CD8+ cells responding to stimulation with TERT357 peptide pool (Supplementary Table S1) in mice DNA immunized with mixture of Core152opt and TERT (MX) compared to vector pVax1 (Mann-Whitney *U* test w/ continuity correction). **Table S6**. Statistical comparison of populations CD4+ and CD8+ cells responding to stimulation with TERT6 and TERT8 peptides pool (Supplementary Table S1) in mice DNA immunized with TERT compared to vector pVax1 (Mann-Whitney *U* test w/ continuity correction). **Table S7**. Statistical comparison of populations CD4+ and CD8+ cells responding to stimulation with TERT6 and TERT8 peptides pool (Supplementary Table S1) in mice DNA immunized with mixture of Core152opt and TERT (MX) compared to vector pVax1 (Mann-Whitney *U* test w/ continuity correction). **Table S8**. Pairwise comparison of the strength of photon flux (p/s) reflecting expression of luciferase from the injection sites in mice receiving boosts of plasmids encoding HCV core (Core191v, Core152opt), TERT and mixture of plasmids Core152opt and TERT (Table 1) together with plasmid encoding luciferase, 24 h post co-delivery.
