# Supplementary material for: Reciprocal Inhibition of Immunogenic Performance in Mice of Two Potent DNA Immunogens Targeting HCV-Related Liver Cancer
Source: Microorganisms. 2021 May 17;9(5):1073. doi: 10.3390/microorganisms9051073 (PMC8156932; doi:10.3390/microorganisms9051073)
Supplement: Supplementary file 1 [file microorganisms-09-01073-s001.zip › microorganisms-1155256-SI.pdf]

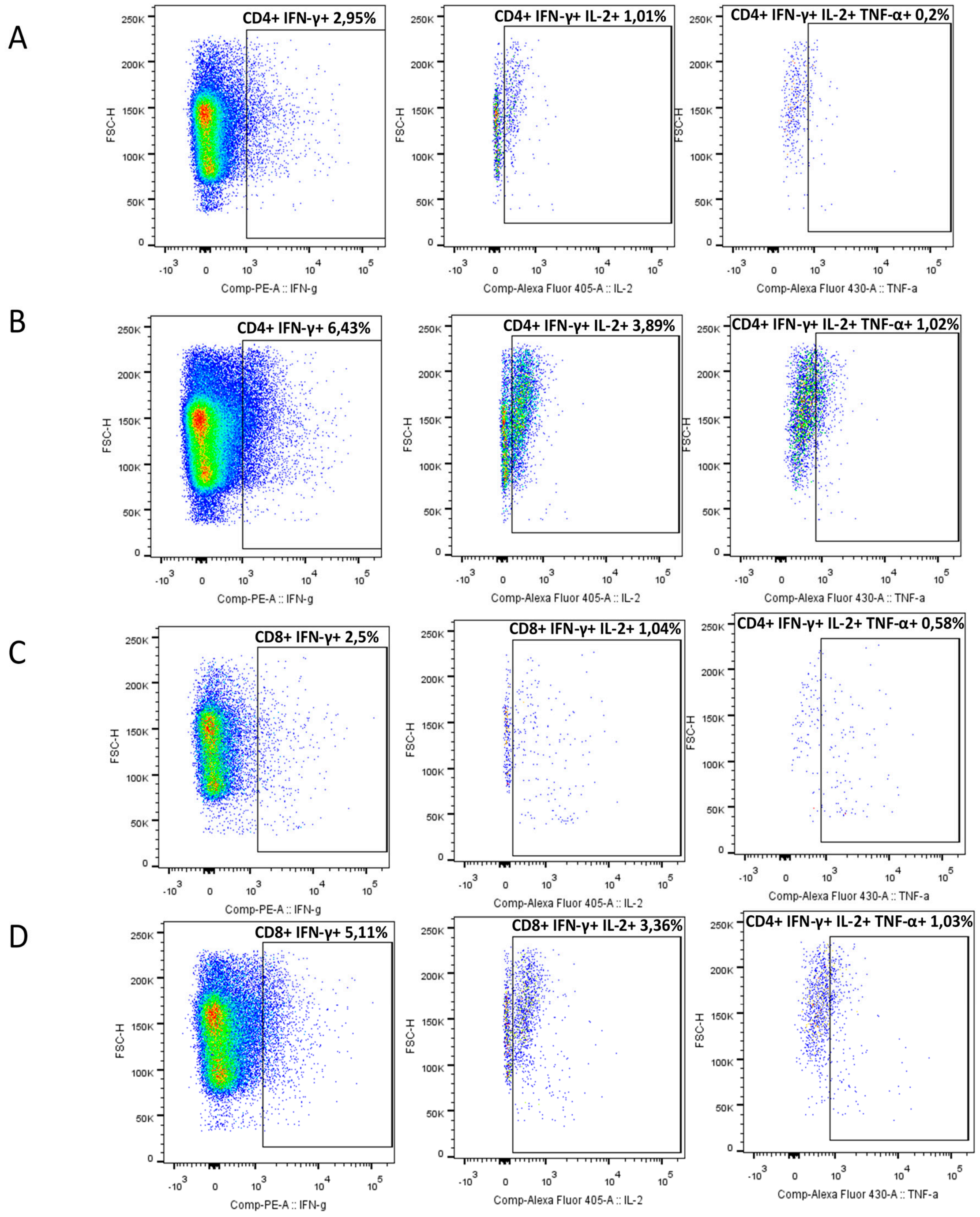

Supplementary Figure S1. Principles of inclusive gating applied in the analysis of specific T-cell response in DNA immunized mice using multiparametric flow cytometry. CD4+ (A, B) and CD8+ (C, D) T cells of mice immunized with pVax1 (A, C) or pVaxCore152opt (B, D) stimulated with poolof peptides derived from HCV core (CORE POOL, Suppl. Table S1). Size of populations

expressing IFN-γ, IFN-γ/IL-2 and IFN-γ/IL-2/TNF-α, based on inclusive principles, is shown as % of the respective CD4+ or CD8+ T cell populations. For immunization details, see Materials and Methods and Table 1.

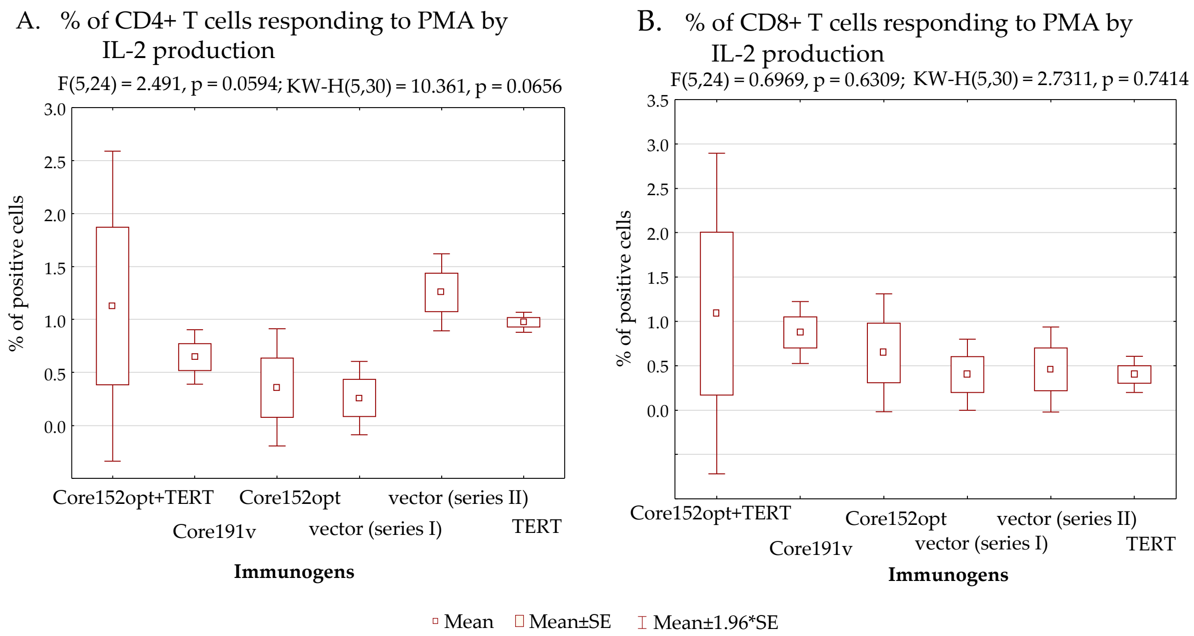

Supplementary Figure S2. Splenocytes of mice in experimental series I and II (Table 1) demonstrate efficient response to stimulation with mitogen(s). In Series I, splenocytes of mice were stimulated with phorbol 12-myristate 13-acetate (PMA; 50 ng/mL), and in Series II, with a mix of PMA and ionomycin (1 µg/mL). IL-2 positive CD4+ (A) and CD8+ (B) T cells in groups of mice DNA immunized with Core191v, Core152opt, empty vector (series I), TERT, Core152opt+TERT and vector (series 2), in %**.** p>0,05, Kruskal Wallis test.

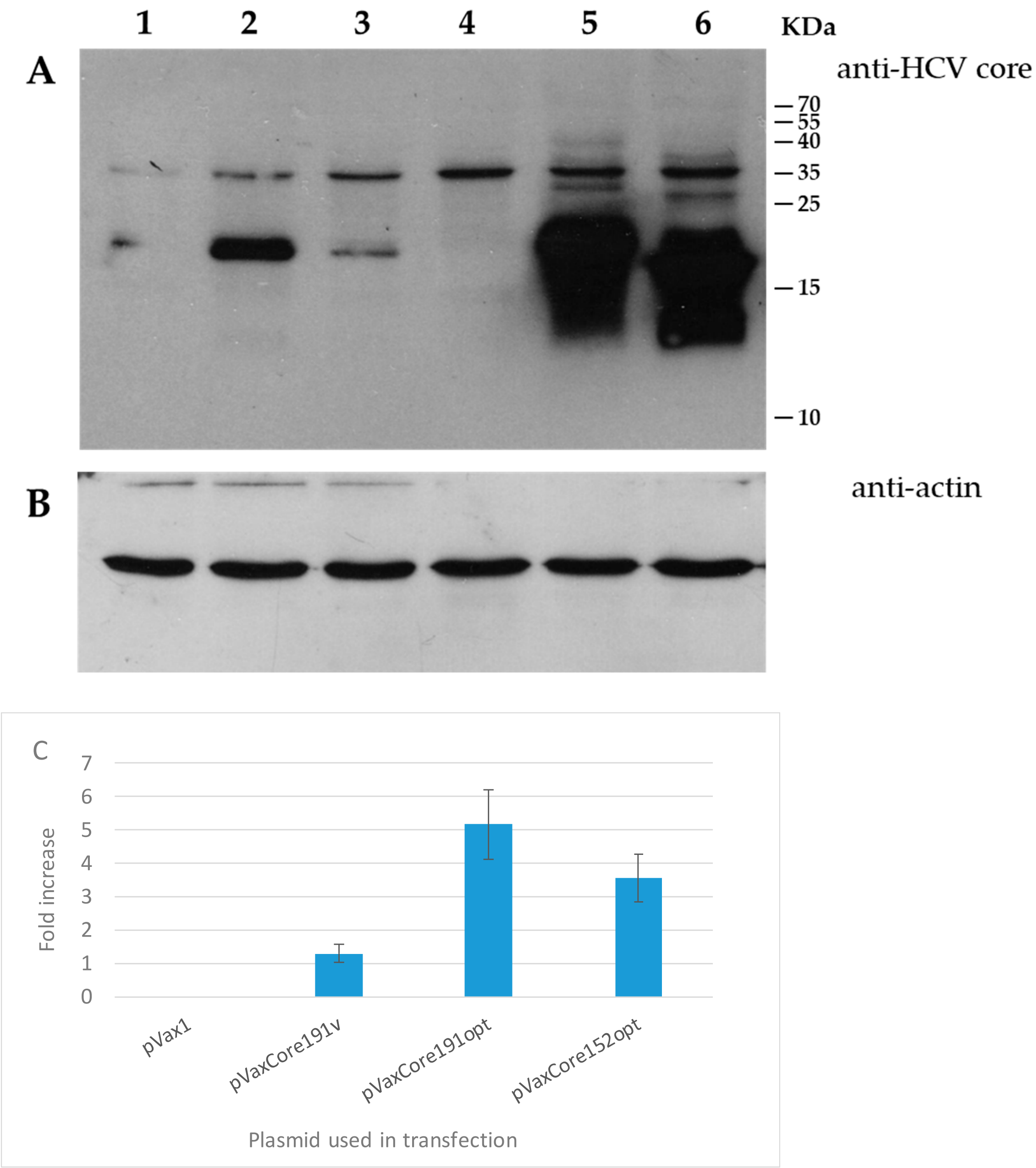

Supplementary Figure S3. Expression of HCV core variants in transiently transfected Huh-7 cells. In brief, Huh-7 cells were transfected with a panel of plasmids pVax1 (lane 1), pVaxCore191v (2), pVaxCore191opt (5), pVaxCore152opt (6) (lanes 3 and 4, transfections with pVaxCore191 plasmid with mutations), for pVaxCore152 in the presence of 5μM MG132; 48 h post transfections cells were lysed and subjected to SDS-PAGE with subsequent Western blotting with polyclonal anti-HCV core (A); blots were stripped and re-stained with anti-actin antibodies (B); HCV core expression

normalized to that of actin (C). Figures to the right represent position of the molecular mass marker PageRuler Plus Prestained Protein Ladder (Thermo Scientific). Signals were quantified using ImageJ. Data represent the results of two independent runs, exhibiting 20% deviation.

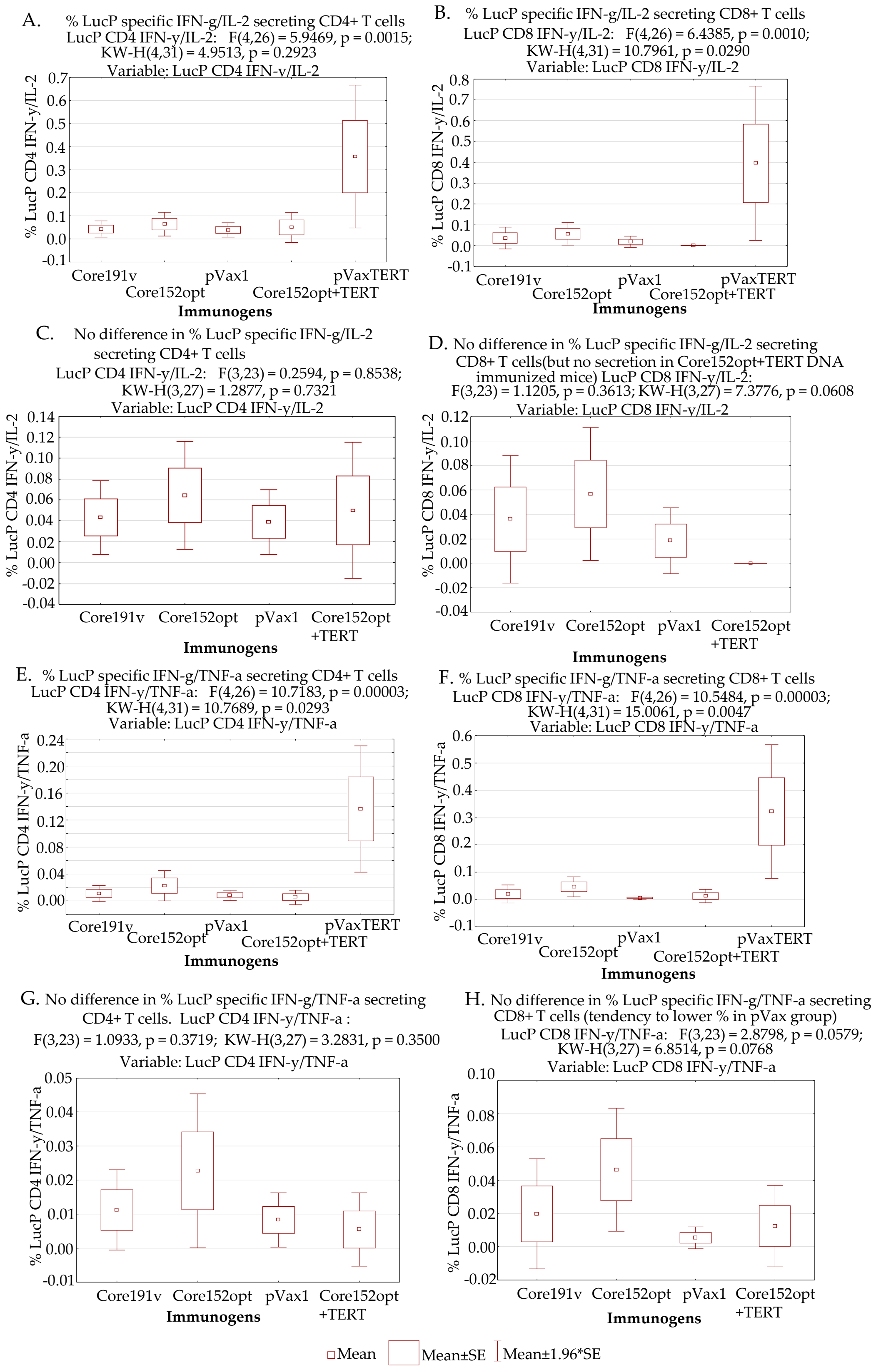
A. % LucP specific IFN-g/IL-2 secreting CD4+ T cells
LucP CD4 IFN-y/IL-2: F(4,26) = 5.9469, p = 0.0015; KW-H(4,31) = 4.9513, p = 0.2923
Variable: LucP CD4 IFN-y/IL-2
% LucP CD4 IFN-y/IL-2
Core191v
Core152opt
pVax1
Core152opt+TERT
pVaxTERT
Immunogens
B. % LucP specific IFN-g/IL-2 secreting CD8+ T cells
LucP CD8 IFN-y/IL-2: F(4,26) = 6.4385, p = 0.0010; KW-H(4,31) = 10.7961, p = 0.0290
Variable: LucP CD8 IFN-y/IL-2
% LucP CD8 IFN-y/IL-2
Core191v
Core152opt
pVax1
Core152opt+TERT
pVaxTERT
Immunogens
C. No difference in % LucP specific IFN-g/IL-2 secreting CD4+ T cells
LucP CD4 IFN-y/IL-2: F(3,23) = 0.2594, p = 0.8538; KW-H(3,27) = 1.2877, p = 0.7321
Variable: LucP CD4 IFN-y/IL-2
% LucP CD4 IFN-y/IL-2
Core191v
Core152opt
pVax1
Core152opt +TERT
Immunogens
D. No difference in % LucP specific IFN-g/IL-2 secreting CD8+ T cells(but no secretion in Core152opt+TERT DNA immunized mice) LucP CD8 IFN-y/IL-2:
F(3,23) = 1.1205, p = 0.3613; KW-H(3,27) = 7.3776, p = 0.0608
Variable: LucP CD8 IFN-y/IL-2
% LucP CD8 IFN-y/IL-2
Core191v
Core152opt
pVax1
Core152opt +TERT
Immunogens
E. % LucP specific IFN-g/TNF-a secreting CD4+ T cells
LucP CD4 IFN-y/TNF-a: F(4,26) = 10.7183, p = 0.00003; KW-H(4,31) = 10.7689, p = 0.0293
Variable: LucP CD4 IFN-y/TNF-a
% LucP CD4 IFN-y/TNF-a
Core191v
Core152opt
pVax1
Core152opt+TERT
pVaxTERT
Immunogens
F. % LucP specific IFN-g/TNF-a secreting CD8+ T cells
LucP CD8 IFN-y/TNF-a: F(4,26) = 10.5484, p = 0.00003; KW-H(4,31) = 15.0061, p = 0.0047
Variable: LucP CD8 IFN-y/TNF-a
% LucP CD8 IFN-y/TNF-a
Core191v
Core152opt
pVax1
Core152opt+TERT
pVaxTERT
Immunogens
G. No difference in % LucP specific IFN-g/TNF-a secreting CD4+ T cells. LucP CD4 IFN-y/TNF-a :
F(3,23) = 1.0933, p = 0.3719; KW-H(3,27) = 3.2831, p = 0.3500
Variable: LucP CD4 IFN-y/TNF-a
% LucP CD4 IFN-y/TNF-a
Core191v
Core152opt
pVax1
Core152opt +TERT
Immunogens
H. No difference in % LucP specific IFN-g/TNF-a secreting CD8+ T cells (tendency to lower % in pVax group)
LucP CD8 IFN-y/TNF-a: F(3,23) = 2.8798, p = 0.0579; KW-H(3,27) = 6.8514, p = 0.0768
Variable: LucP CD8 IFN-y/TNF-a
% LucP CD8 IFN-y/TNF-a
Core191v
Core152opt
pVax1
Core152opt +TERT
Immunogens
Mean
Mean±SE
Mean±1.96*SE

Supplementary Figure S4. Statistical comparison of T cell response to immunodominant epitope of luciferase LucP in mice DNA immunized with Core191v, Core152opt, TERT, mixture of Core152opt and TERT and empty vector. Data from immunization series I and II; for vector mice represent combined data from series I and II (Table 1). Percent CD4+ (A, C) and CD8+ T cells (B, D) secreting IFN-γ/IL-2 in all groups (A, B) and in groups excluding TERT immunized mice (C, D); Percent CD4+ (E, G) and CD8+ T cells (F, H) secreting IFN-γ/TNF-α in all groups (E, F) and in groups excluding TERT immunized mice (G, H); Percent of cytokine secreting CD4+ and CD8+ T cells is represented by mean ± standard error (SE). No difference between the groups after exclusion of mice immunized with TERT DNA (C, D, G, H) in Kruskal Wallis test, and in pair-wise comparisons, Mann-Whitney U-test (Statistica Tibco, version 13.5).

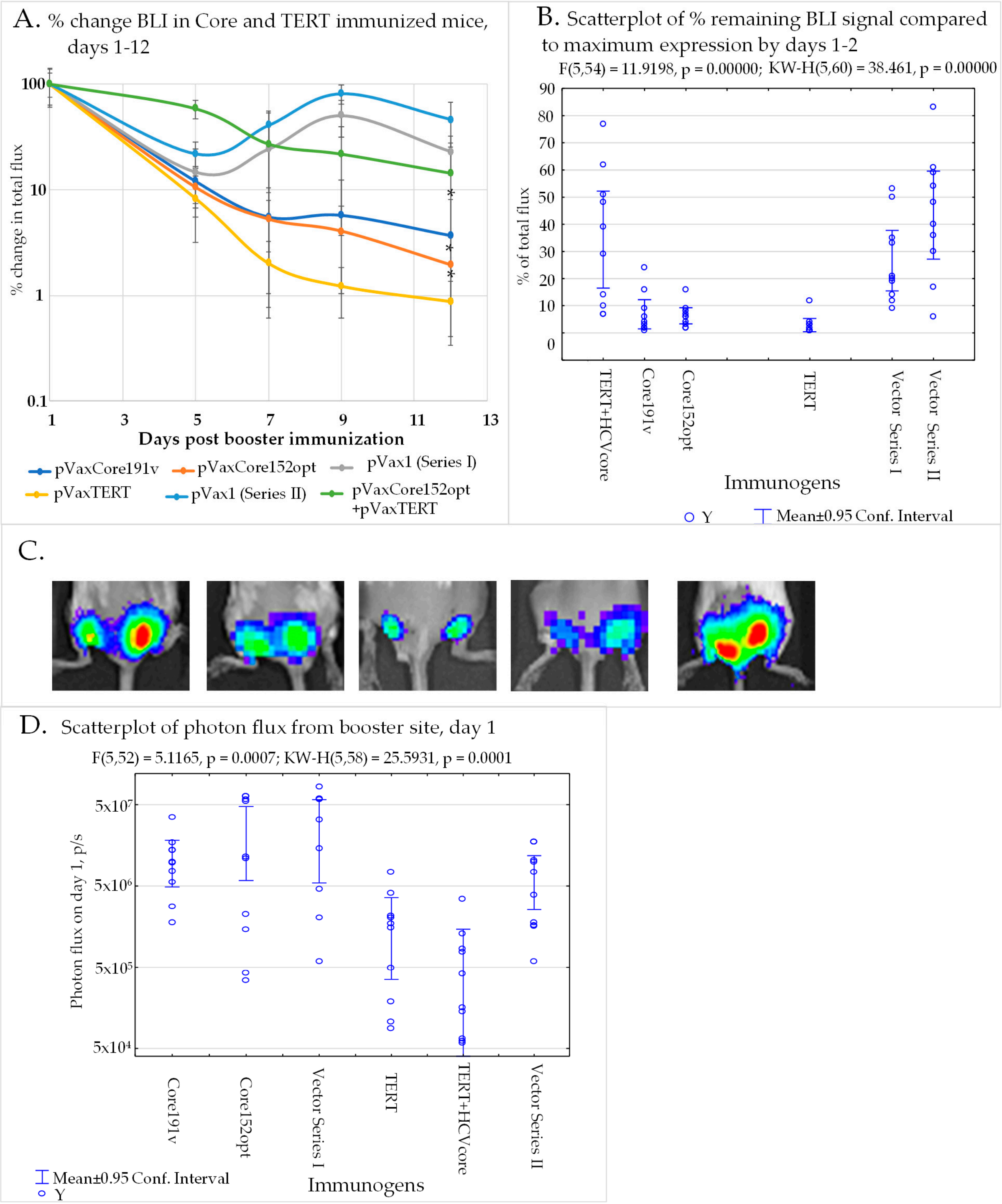

Supplementary Figure S5. Bioluminescence from DNA booster sites in mice receiving HCV core and TERT alone or in a mix. Groups of BALB/c mice (n=5) were DNA-immunized with Core191v, Core152opt, TERT, Core152opt and TERT mixture (TERT+Core152opt) and empty vector (Series I and II; Table 1). Change in BLI signal from DNA booster sites from day 1 to 12, in % (A); %

remaining BLI signal by day 7 after the boost compared to maximal expression by days 1-2 (B); Individual mice 24 h after receiving booster injections with pVaxLuc2 mixed with pVaxCore191v (1), pVaxCore152opt (2), pVaxTERT (3), pVaxCore152opt/pVaxTERT (4), pVax1 (5) administered to the left and to the right from the base of the tail (C); Scatterplot of BLI from all injection sites one day after the boost (D). Average ± STDV per immunization site (A); individual booster sites (B, C); mean ± error (0.95 confidence interval) per immunization site (D) * - $p<0,05$ between Core191v, Core152opt, and TERT DNA immunized compared to vector immunized mice starting from day 7, Mann Whitney U-test (A); $p<0,000001$, % BLI change by day 7, Kruskal-Wallis test (B).

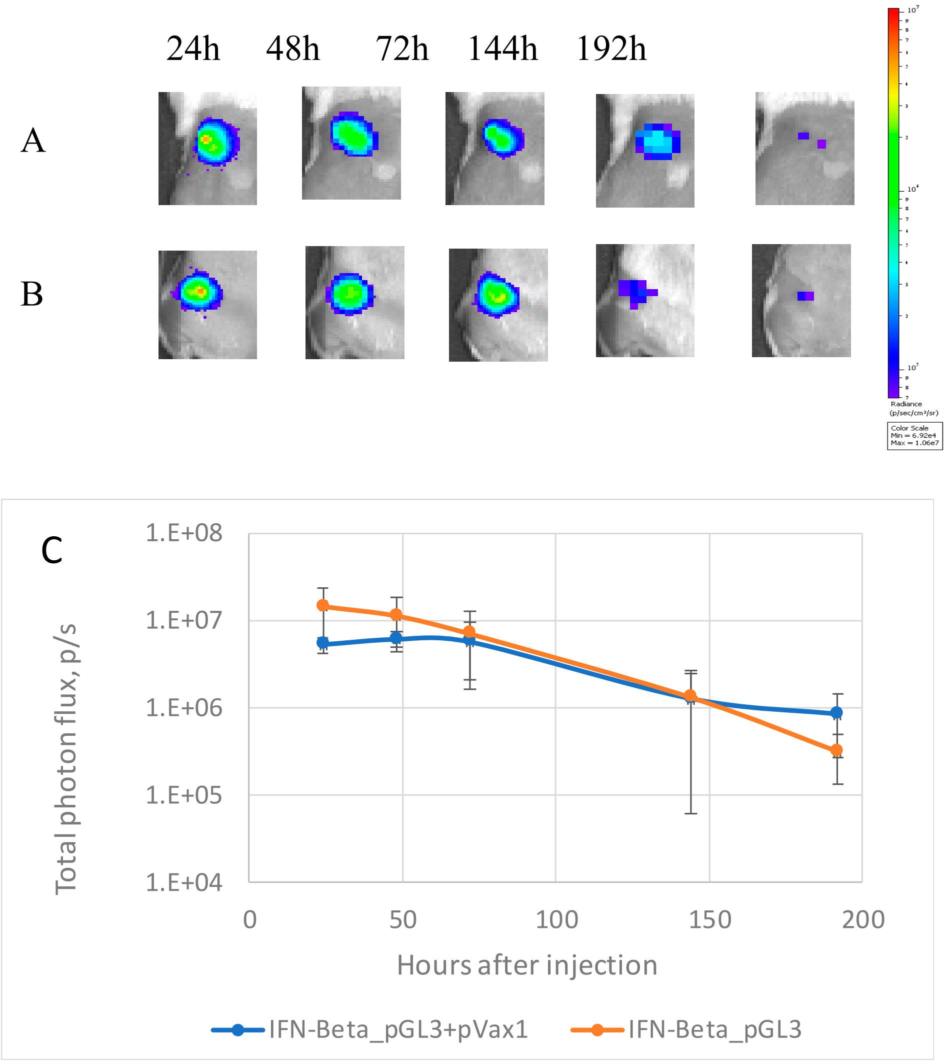

Supplementary Figure S6. Expression in mice of luciferase reporter from the IFN-b promoter. *In vivo* bioluminescent imaging of mice receiving IFN-Beta_pGL3 reporter plasmid (10 mg per site) (A), or IFN-Beta_pGL3 mixed with pVax1 (10 mg of each per site) (B) monitored 24, 48, 72, 144 and 196 h after the injection; Kinetics of the gradual loss of BLI signal, mean ± STDV (C). Signal intensity in photons/sec is represented as a color scale to the right of the images.

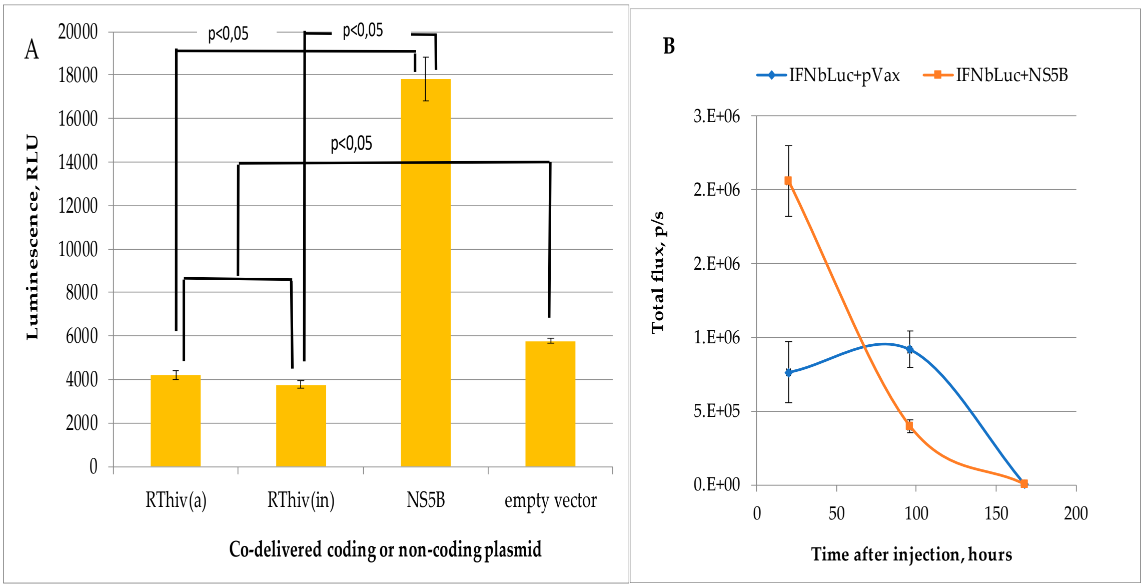

Supplementary Figure S7. Expression of luciferase reporter from the IFN-β promoter *in vitro* (A) and *in vivo* (B). Luminescence signal (in RLU) emitted by the lysates of HEK293 cells co-transfected with IFN-Beta_pGL3 reporter plasmid and pVax1 vector, or plasmid encoding dependent RNA-polymerase of HCV (NS5B), or enzymatically active or inactivated HIV-1 reverse transcriptase, RThiv(a) and RThiv(in), respectively (see Materials and methods for details (A); Total photon flux (mean ± STDV) from the sites of injection into mice of IFN-Beta_pGL3 reporter mixed with pVax1 vector, or plasmid encoding dependent RNA-polymerase of HCV (NS5B) (10 mg of each per site) registered by *in vivo* BLI of mice (n=2) on days 1, 4 and 7 after the injections (B). * - p<0,05, Mann-Whitney U test.

Supplementary Table S1. Synthetic peptides used in the study.

Protein	Position, first/last amino acid residue	Amino acid sequence	Abbreviation
HCV core	61-95	RRQPIPKARRPEGRTWAQPGYPWPLYGNEGMGWAG	CORE POOL
	81-115	YPWPLYGNEGMGWAGWLLSPRGSRPSWGPNDPRRR	
	101-135	RGSRPSWGPNDPRRRSRNLGKVIDTLTCGFADLMG	
	141-175	GAPLGGAARALAHGVRVLEDGVNYATGNLPGCSFS	
TERT	559-584	QKNRLFFYRKSVWSKLQSIGIRQQL	TERT357 (pool)
	791-815	SLLHFFLRFVRHSVVKIDGRFYVQ	
	888-917	KTVVNFPVETGALGGAAPHQLPAHCLFPW	
	845-865	QQDGLLLRFVDDFLLVTPHL	TERT6
	901-929	LGGAAPHQLPAHCLFPWCGLLLDTRTLE	TERT8
Firefly luciferase	160-168	GFQSMYTFV	LucP

Supplementary Table S2. Statistical comparison of populations CD4+ and CD8+ cells responding to stimulation with HCV core peptide pool (Supplementary Table S1) in mice DNA immunized with mixture of Core152opt and TERT (MIX) comparing to vector pVax1 (Mann-Whitney U test w/ continuity correction).

	Rank Sum (MIX)	Rank Sum (vector)	U	Z	p-value	Z (adjusted)	p-value	Valid N (MIX)	Valid N (vector)	2*1sided (exact p)
CD4 IFN-y	36.00000	55.00000	10.00000	1.15728	0.247161	1.56517	0.117545	4	9	0.260140
CD4 IL-2	37.00000	54.00000	9.00000	1.31158	0.189663	2.08528	0.037045	4	9	0.198601
CD4 TNF-a	25.00000	66.00000	15.00000	-0.38576	0.699676	-0.47116	0.637525	4	9	0.710490
CD4 IFN-y/IL-2	35.00000	56.00000	11.00000	1.00297	0.315875	1.35648	0.174948	4	9	0.330070
CD4 IFN-y/TNF-a	40.00000	51.00000	6.00000	1.77449	0.075983	2.16735	0.030209	4	9	0.075524
CD4 IL-2/TNF-a	35.00000	56.00000	11.00000	1.00297	0.315875	1.35648	0.174948	4	9	0.330070
CD4 IFN-y/IL-2/TNF-a	41.00000	50.00000	5.00000	1.92879	0.053758	2.35581	0.018483	4	9	0.050350
CD8 IFN-y	32.00000	59.00000	14.00000	0.54006	0.589155	0.61577	0.538050	4	9	0.604196
CD8 IL-2	31.00000	60.00000	15.00000	0.38576	0.699676	0.61332	0.539667	4	9	0.710490
CD8 TNF-a	25.00000	66.00000	15.00000	-0.38576	0.699676	-0.47116	0.637525	4	9	0.710490
CD8 IFN-y/IL-2	25.00000	66.00000	15.00000	-0.38576	0.699676	-0.47116	0.637525	4	9	0.710490
CD8 IFN-y/TNF-a	28.50000	62.50000	17.50000	0.00000	1.000000	0.00000	1.000000	4	9	0.939860
CD8 IL-2/TNF-a	26.50000	64.50000	16.50000	-0.15430	0.877371	-0.17593	0.860347	4	9	0.825175
CD8 IFN-y/IL-2/TNF-a	28.00000	63.00000	18.00000	0.07715	0.938503	0.09423	0.924924	4	9	1.000000

Supplementary Table S3. Statistical comparison of populations CD4+ and CD8+ cells responding to stimulation with HCV core peptide pool (Supplementary S1) in mice DNA immunized with mixture of Core152opt and TERT (MIX) comparing to Core152opt (Mann-Whitney U test w/ continuity correction).

	Rank Sum (MIX)	Rank Sum (Core152opt)	U	Z	p-value	Z (adjusted)	p-value	Valid N (MIX)	Valid N (Core152opt)	2*1sided (exact p)
CD4 IFN-y	19.50000	25.50000	9.500000	0.00000	1.000000	0.00000	1.000000	4	5	0.904762
CD4 IL-2	10.00000	35.00000	0.000000	-2.32702	0.019965	-2.33677	0.019452	4	5	0.015873
CD4 TNF-a	12.50000	32.50000	2.500000	-1.71464	0.086412	-1.79089	0.073313	4	5	0.063492
CD4 IFN-y/IL-2	13.00000	32.00000	3.000000	-1.59217	0.111348	-1.59884	0.109856	4	5	0.111111
CD4 IFN-y/TNF-a	17.50000	27.50000	7.500000	-0.48990	0.624206	-0.49195	0.622754	4	5	0.555556
CD4 IL-2/TNF-a	16.00000	29.00000	6.000000	-0.85732	0.391268	-0.86092	0.389285	4	5	0.412698
CD4 IFN-y/IL-2/TNF-a	14.00000	31.00000	4.000000	-1.34722	0.177911	-1.34722	0.177911	4	5	0.190476
CD8 IFN-y	14.00000	31.00000	4.000000	-1.34722	0.177911	-1.35287	0.176099	4	5	0.190476
CD8 IL-2	11.00000	34.00000	1.000000	-2.08207	0.037337	-2.11766	0.034205	4	5	0.031746
CD8 TNF-a	15.00000	30.00000	5.000000	-1.10227	0.270345	-1.20748	0.227250	4	5	0.285714
CD8 IFN-y/IL-2	10.00000	35.00000	0.000000	-2.32702	0.019965	-2.36680	0.017943	4	5	0.015873
CD8 IFN-y/TNF-a	10.00000	35.00000	0.000000	-2.32702	0.019965	-2.36680	0.017943	4	5	0.015873
CD8 IL-2/TNF-a	15.50000	29.50000	5.500000	-0.97980	0.327188	-1.07872	0.280713	4	5	0.285714
CD8 IFN-y/IL-2/TNF-a	10.00000	35.00000	0.000000	-2.32702	0.019965	-2.36680	0.017943	4	5	0.015873

Supplementary Table S4. Statistical comparison of populations CD4+ and CD8+ cells responding to stimulation with TERT357 peptide pool (Supplementary Table S1) in mice DNA immunized with TERT comparing to vector pVax1 (Mann-Whitney U test w/ continuity correction).

T cell populations	Rank Sum (TERT)	Rank Sum (vector)	U	Z	p-value	Z (adjusted)	p-value	Valid N (TERT)	Valid N (vector)	2*1sided (exact p)
CD4 IFN-y	55.00000	50.00000	5.00000	2.266667	0.023411	2.269162	0.023259	5	9	0.018981
CD4 IL-2	57.00000	48.00000	3.00000	2.533333	0.011299	2.536122	0.011209	5	9	0.006993
CD4 TNF-a	43.00000	62.00000	17.00000	0.666667	0.504986	0.674116	0.500238	5	9	0.518482
CD4 IFN-y/IL-2	59.00000	46.00000	1.00000	2.800000	0.005111	2.803082	0.005062	5	9	0.001998
CD4 IFN-y/TNF-a	57.00000	48.00000	3.00000	2.533333	0.011299	2.547369	0.010854	5	9	0.006993
CD4 IL-2/TNF-a	54.00000	51.00000	6.00000	2.133333	0.032898	2.142773	0.032132	5	9	0.028971
CD4 IFN-y/IL-2/TNF-a	58.00000	47.00000	2.00000	2.666667	0.007661	2.669602	0.007595	5	9	0.003996
CD8 IFN-y	54.00000	51.00000	6.00000	2.133333	0.032898	2.138037	0.032514	5	9	0.028971
CD8 IL-2	56.00000	49.00000	4.00000	2.400000	0.016396	2.402642	0.016278	5	9	0.011988
CD8 TNF-a	45.00000	60.00000	15.00000	0.933333	0.350649	0.943762	0.345292	5	9	0.363636
CD8 IFN-y/IL-2	58.00000	47.00000	2.00000	2.666667	0.007661	2.669602	0.007595	5	9	0.003996
CD8 IFN-y/TNF-a	58.00000	47.00000	2.00000	2.666667	0.007661	2.678466	0.007396	5	9	0.003996
CD8 IL-2/TNF-a	59.00000	46.00000	1.00000	2.800000	0.005111	2.812389	0.004918	5	9	0.001998
CD8 IFN-y/IL-2/TNF-a	58.00000	47.00000	2.00000	2.666667	0.007661	2.678466	0.007396	5	9	0.003996

Supplementary Table S5. Statistical comparison of populations CD4+ and CD8+ cells responding to stimulation with TERT357 peptide pool (Supplementary S1) in mice DNA immunized with mixture of Core152opt and TERT (MX) comparing to vector pVax1 (Mann-Whitney U test w/ continuity correction).

	Rank Sum (MIX)	Rank Sum (vector)	U	Z	p-value	Z (adjusted)	p-value	Valid N (MIX)	Valid N (vector)	2*1sided (exact p)
CD4 IFN-y	22.00000	69.00000	12.00000	-0.84867	0.396066	-0.85337	0.393455	4	9	0.413986
CD4 IL-2	23.00000	68.00000	13.00000	-0.69437	0.487454	-0.69821	0.485045	4	9	0.503497
CD4 TNF-a	24.00000	67.00000	14.00000	-0.54006	0.589155	-0.56806	0.569993	4	9	0.604196
CD4 IFN-y/IL-2	23.50000	67.50000	13.50000	-0.61721	0.537094	-0.62150	0.534273	4	9	0.503497
CD4 IFN-y/TNF-a	24.00000	67.00000	14.00000	-0.54006	0.589155	-0.55635	0.577973	4	9	0.604196
CD4 IL-2/TNF-a	23.00000	68.00000	13.00000	-0.69437	0.487454	-0.71426	0.475064	4	9	0.503497
CD4 IFN-y/IL-2/TNF-a	24.00000	67.00000	14.00000	-0.54006	0.589155	-0.54764	0.583942	4	9	0.604196
CD8 IFN-y	19.00000	72.00000	9.00000	-1.31158	0.189663	-1.31884	0.187222	4	9	0.198601
CD8 IL-2	24.00000	67.00000	14.00000	-0.54006	0.589155	-0.54764	0.583942	4	9	0.604196
CD8 TNF-a	24.00000	67.00000	14.00000	-0.54006	0.589155	-0.56806	0.569993	4	9	0.604196
CD8 IFN-y/IL-2	20.00000	71.00000	10.00000	-1.15728	0.247161	-1.17351	0.240593	4	9	0.260140
CD8 IFN-y/TNF-a	22.00000	69.00000	12.00000	-0.84867	0.396066	-0.87299	0.382669	4	9	0.413986
CD8 IL-2/TNF-a	24.50000	66.50000	14.50000	-0.46291	0.643429	-0.47687	0.633455	4	9	0.604196
CD8 IFN-y/IL-2/TNF-a	22.00000	69.00000	12.00000	-0.84867	0.396066	-0.87299	0.382669	4	9	0.413986

Supplementary Table S6. Statistical comparison of populations CD4+ and CD8+ cells responding to stimulation with TERT6 and TERT8 peptides pool (Supplementary Table S1) in mice DNA immunized with TERT comparing to vector pVax1 (Mann-Whitney U test w/ continuity correction).

T cell population	Rank Sum (TERT)	Rank Sum (vector)	U	Z	p-value	Z (adjusted)	p-value	Valid N (MIX)	Valid N (vector)	2*1sided (exact p)
TERT6 CD4 IFN-y	48.00000	57.00000	12.00000	1.333333	0.182423	1.425617	0.153980	5	9	0.189810
TERT6 CD4 IL-2	48.00000	57.00000	12.00000	1.333333	0.182423	1.423830	0.154497	5	9	0.189810
TERT6 CD4 TNF-a	35.00000	70.00000	20.00000	-0.266667	0.789726	-0.596285	0.550985	5	9	0.797203
TERT6 CD4 IFN-y/IL-2	47.50000	57.50000	12.50000	1.266667	0.205275	1.354336	0.175630	5	9	0.189810
TERT6 CD4 IFN-y/TNF-a	48.00000	57.00000	12.00000	1.333333	0.182423	1.423830	0.154497	5	9	0.189810
TERT6 CD4 IL-2/TNF-a	51.50000	53.50000	8.50000	1.800000	0.071862	1.924583	0.054282	5	9	0.059940
TERT6 CD4 IFN-y/IL-2/TNF-a	48.50000	56.50000	11.50000	1.400000	0.161514	1.496898	0.134421	5	9	0.146853
TERT6 CD8 IFN-y	50.00000	55.00000	10.00000	1.600000	0.109600	1.708595	0.087527	5	9	0.111888
TERT6 CD8 IL-2	54.50000	50.50000	5.50000	2.200000	0.027808	2.436360	0.014836	5	9	0.018981
TERT6 CD8 TNF-a	47.00000	58.00000	13.00000	1.200000	0.230140	1.503100	0.132814	5	9	0.239760
TERT6 CD8 IFN-y/IL-2	54.00000	51.00000	6.00000	2.133333	0.032898	2.278127	0.022720	5	9	0.028971
TERT6 CD8 IFN-y/TNF-a	55.00000	50.00000	5.00000	2.266667	0.023411	2.641624	0.008251	5	9	0.018981
TERT6 CD8 IL-2/TNF-a	55.50000	49.50000	4.50000	2.333333	0.019631	2.922695	0.003470	5	9	0.011988
TERT6 CD8 IFN-y/IL-2/TNF-a	55.00000	50.00000	5.00000	2.266667	0.023411	2.641624	0.008251	5	9	0.018981
TERT8 CD4 IFN-y	52.50000	52.50000	7.50000	1.93333	0.053196	1.95714	0.050332	5	9	0.041958
TERT8 CD4 IL-2	53.00000	52.00000	7.00000	2.00000	0.045501	2.14653	0.031831	5	9	0.041958
TERT8 CD4 TNF-a	36.00000	69.00000	21.00000	-0.13333	0.893930	-0.18553	0.852815	5	9	0.898102
TERT8 CD4 IFN-y/IL-2	54.00000	51.00000	6.00000	2.13333	0.032898	2.28099	0.022550	5	9	0.028971
TERT8 CD4 IFN-y/TNF-a	52.00000	53.00000	8.00000	1.86667	0.061949	1.90910	0.056251	5	9	0.059940
TERT8 CD4 IL-2/TNF-a	52.00000	53.00000	8.00000	1.86667	0.061949	1.99586	0.045950	5	9	0.059940
TERT8 CD4 IFN-y/IL-2/TNF-a	52.00000	53.00000	8.00000	1.86667	0.061949	1.99336	0.046223	5	9	0.059940
TERT8 CD8 IFN-y	51.50000	53.50000	8.50000	1.80000	0.071862	1.80997	0.070301	5	9	0.059940
TERT8 CD8 IL-2	43.00000	62.00000	17.00000	0.66667	0.504986	0.68182	0.495353	5	9	0.518482
TERT8 CD8 TNF-a	29.00000	76.00000	14.00000	-1.06667	0.286123	-1.18127	0.237498	5	9	0.297702
TERT8 CD8 IFN-y/IL-2	46.50000	58.50000	13.50000	1.13333	0.257075	1.14600	0.251797	5	9	0.239760
TERT8 CD8 IFN-y/TNF-a	46.00000	59.00000	14.00000	1.06667	0.286123	1.09091	0.275312	5	9	0.297702
TERT8 CD8 IL-2/TNF-a	42.50000	62.50000	17.50000	0.60000	0.548507	0.61014	0.541768	5	9	0.518482
TERT8 CD8 IFN-y/IL-2/TNF-a	51.00000	54.00000	9.00000	1.73333	0.083037	1.77273	0.076274	5	9	0.082917

Supplementary Table S7. Statistical comparison of populations CD4+ and CD8+ cells responding to stimulation with TERT6 and TERT8 peptides pool (Supplementary Table S1) in mice DNA immunized with mixture of Core152opt and TERT (MX) comparing to vector pVax1 (Mann-Whitney U test w/ continuity correction).

T cell population	Rank Sum (MIX)	Rank Sum (vector)	U	Z	p-value	Z (adjusted)	p-value	Valid N (MIX)	Valid N (vector)	2*1sided (exact p)
TERT6 CD4 IFN-y	29.00000	62.00000	17.00000	0.07715	0.938503	0.08797	0.929903	4	9	0.939860
TERT6 CD4 IL-2	26.00000	65.00000	16.00000	-0.23146	0.816961	-0.28270	0.777409	4	9	0.825175
TERT6 CD4 TNF-a	26.00000	65.00000	16.00000	-0.23146	0.816961	-0.50000	0.617075	4	9	0.825175
TERT6 CD4 IFN-y/IL-2	30.00000	61.00000	16.00000	0.23146	0.816961	0.26390	0.791858	4	9	0.825175
TERT6 CD4 IFN-y/TNF-a	26.00000	65.00000	16.00000	-0.23146	0.816961	-0.28270	0.777409	4	9	0.825175
TERT6 CD4 IL-2/TNF-a	26.00000	65.00000	16.00000	-0.23146	0.816961	-0.28270	0.777409	4	9	0.825175
TERT6 CD4 IFN-y/IL-2/TNF-a	26.00000	65.00000	16.00000	-0.23146	0.816961	-0.28270	0.777409	4	9	0.825175
TERT6 CD8 IFN-y	28.00000	63.00000	18.00000	0.07715	0.938503	0.09423	0.924924	4	9	1.000000
TERT6 CD8 IL-2	29.50000	61.50000	16.50000	0.15430	0.877371	0.20869	0.834691	4	9	0.825175
TERT6 CD8 TNF-a	31.00000	60.00000	15.00000	0.38576	0.699676	0.61332	0.539667	4	9	0.710490
TERT6 CD8 IFN-y/IL-2	28.00000	63.00000	18.00000	-0.07715	0.938503	-0.09423	0.924924	4	9	1.000000
TERT6 CD8 IFN-y/TNF-a	31.00000	60.00000	15.00000	0.38576	0.699676	0.61332	0.539667	4	9	0.710490
TERT6 CD8 IL-2/TNF-a	32.50000	58.50000	13.50000	0.61721	0.537094	1.33333	0.182423	4	9	0.503497
TERT6 CD8 IFN-y/IL-2/TNF-a	31.00000	60.00000	15.00000	0.38576	0.699676	0.61332	0.539667	4	9	0.710490
TERT8 CD4 IFN-y	31.00000	60.00000	15.00000	0.385758	0.699676	0.397392	0.691079	4	9	0.710490
TERT8 CD4 IL-2	33.00000	58.00000	13.00000	0.694365	0.487454	0.791698	0.428537	4	9	0.503497
TERT8 CD4 TNF-a	27.50000	63.50000	17.50000	0.000000	1.000000	0.000000	1.000000	4	9	0.939860
TERT8 CD4 IFN-y/IL-2	33.50000	57.50000	12.50000	0.771517	0.440401	0.881239	0.378189	4	9	0.413986
TERT8 CD4 IFN-y/TNF-a	26.50000	64.50000	16.50000	-0.154303	0.877371	-0.168018	0.866569	4	9	0.825175
TERT8 CD4 IL-2/TNF-a	28.00000	63.00000	18.00000	0.077152	0.938503	0.088124	0.929778	4	9	1.000000
TERT8 CD4 IFN-y/IL-2/TNF-a	34.00000	57.00000	12.00000	0.848668	0.396066	0.922600	0.356216	4	9	0.413986
TERT8 CD8 IFN-y	16.00000	75.00000	6.00000	-1.774489	0.075983	-1.825344	0.067950	4	9	0.075524
TERT8 CD8 IL-2	23.00000	68.00000	13.00000	-0.694365	0.487454	-0.754854	0.450337	4	9	0.503497
TERT8 CD8 TNF-a	18.00000	73.00000	8.00000	-1.465882	0.142681	-1.671362	0.094651	4	9	0.148252
TERT8 CD8 IFN-y/IL-2	19.50000	71.50000	9.50000	-1.234427	0.217045	-1.298429	0.194141	4	9	0.198601
TERT8 CD8 IFN-y/TNF-a	21.00000	70.00000	11.00000	-1.002972	0.315875	-1.090345	0.275562	4	9	0.330070
TERT8 CD8 IL-2/TNF-a	20.00000	71.00000	10.00000	-1.157275	0.247161	-1.258091	0.208360	4	9	0.260140
TERT8 CD8 IFN-y/IL-2/TNF-a	23.50000	67.50000	13.50000	-0.617213	0.537094	-0.703732	0.481600	4	9	0.503497

Supplementary Table S8. Pair-wise comparison of the strength of photon flux (p/s) reflecting expression of luciferase from the injection sites in mice receiving boosts of plasmids encoding HCV core (Core191v, Core152opt), TERT and mixture of plasmids Core152opt and TERT (Table 1) together with plasmid encoding luciferase, 24 hours post co-delivery. Mann-Whitney test (w/continuity correction); marked tests are significant at $p<0.05$.

RANK SUM Group of comparison A	RANK SUM Group of comparison B	U	Z	p-value	Z (adjusted)	p-value	Valid N (Group A)	Valid N (Group B)	2*1sided (exact p)
TERT	TERT+Core152opt								
128.0000	82.00000	27.00000	1.700840	0.088974	1.700840	0.088974	10	10	0.089210
Core191v									
153.0000	57.00000	2.000000	3.590662	0.000330	3.590662	0.000330	10	10	0.000043
Core152opt									
144.0000	66.00000	11.00000	2.910326	0.003611	2.910326	0.003611	10	10	0.002089
Vector Series I									
111.0000	60.00000	5.000000	3.065405	0.002174	3.065405	0.002174	8	10	0.000868
Vector Series II									
148.0000	62.00000	7.000000	3.212698	0.001315	3.212698	0.001315	10	10	0.000487
TERT	Core191v								
62.00000	148.0000	7.000000	-3.21270	0.001315	-3.21270	0.001315	10	10	0.000487
	Core152opt								
77.00000	133.0000	22.00000	-2.07880	0.037636	-2.07880	0.037636	10	10	0.035463
	Vector Series I								
66.00000	87.00000	11.00000	-2.29337	0.021828	-2.29337	0.021828	10	7	0.018511
	Vector Series II								
78.00000	132.0000	23.00000	-2.00321	0.045155	-2.00321	0.045155	10	10	0.043257
Vector Series I	Core191v								
85.00000	86.00000	30.00000	-0.844097	0.398616	-0.844097	0.398616	10	8	0.408245
Vector Series II									
119.0000	91.00000	36.00000	1.020504	0.307490	1.020504	0.307490	10	10	0.314999
Vector Series I	Core152opt								
89.00000	82.00000	34.00000	-0.488688	0.625063	-0.488688	0.625063	10	8	0.633438
Vector Series II									
116.0000	94.00000	39.00000	0.793725	0.427356	0.793725	0.427356	10	10	0.435872
